# Biomolecular Mechanisms of *Pseudomonas aeruginosa* and *Escherichia coli* Biofilm Formation

**DOI:** 10.3390/pathogens3030596

**Published:** 2014-07-18

**Authors:** Garry Laverty, Sean P. Gorman, Brendan F. Gilmore

**Affiliations:** Biomaterials, Biofilm and Infection Control Research Group, School of Pharmacy, Queen’s University Belfast, Medical Biology Centre, 97 Lisburn Road, Belfast BT9 7BL, UK; E-Mails: s.gorman@qub.ac.uk (S.P.G.); b.gilmore@qub.ac.uk (B.F.G.)

**Keywords:** bacteria, biofilm, biomaterial, Gram-negative, infection, quorum sensing

## Abstract

*Pseudomonas aeruginosa* and *Escherichia coli* are the most prevalent Gram-negative biofilm forming medical device associated pathogens, particularly with respect to catheter associated urinary tract infections. In a similar manner to Gram-positive bacteria, Gram-negative biofilm formation is fundamentally determined by a series of steps outlined more fully in this review, namely adhesion, cellular aggregation, and the production of an extracellular polymeric matrix. More specifically this review will explore the biosynthesis and role of pili and flagella in Gram-negative adhesion and accumulation on surfaces in *Pseudomonas aeruginosa* and *Escherichia coli*. The process of biofilm maturation is compared and contrasted in both species, namely the production of the exopolysaccharides via the polysaccharide synthesis locus (*Psl*), pellicle Formation (*Pel*) and alginic acid synthesis in *Pseudomonas aeruginosa,* and UDP-4-amino-4-deoxy-l-arabinose and colonic acid synthesis in *Escherichia coli*. An emphasis is placed on the importance of the LuxR homologue *sdiA*; the *luxS*/autoinducer-II; an autoinducer-III/epinephrine/norepinephrine and indole mediated Quorum sensing systems in enabling Gram-negative bacteria to adapt to their environments. The majority of Gram-negative biofilms consist of polysaccharides of a simple sugar structure (either homo- or heteropolysaccharides) that provide an optimum environment for the survival and maturation of bacteria, allowing them to display increased resistance to antibiotics and predation.

## 1. Introduction

*Pseudomonas aeruginosa* and *Escherichia coli* are the most prevalent Gram-negative biofilm forming medical device associated pathogens [1,2]. Nosocomial infections are estimated to occur annually in 1.75 million hospitalized patients throughout Europe, resulting in 175,000 deaths [3]. *Pseudomonas aeruginosa* accounts for 10%–20% of all hospital-acquired infections [4]. *Pseudomonas aeruginosa* is notoriously difficult to eradicate when colonizing the lungs of cystic fibrosis patients, forming thick antibiotic resistant biofilms that also guard from host immune defenses, lowering of the long-term prognosis of the infected patient [5]. *Escherichia coli* is the most frequently implicated bacteria in urinary catheter related infections, accounting for 50% of such all infections [6,7]. Urinary catheter related infections are the most common form of nosocomial infection with over one million cases a year in the United States alone [7]. In a similar manner to Gram-positive bacteria [8], Gram-negative biofilm formation is determined by the processes of adhesion, cellular aggregation, and the production of an extracellular polymeric matrix with the majority of Gram-negative polysaccharides having a simple structure consisting of either homo- or heteropolysaccharides [9]. The following review will highlight the importance of these stages, and their control at a molecular level, in the production of highly antimicrobial resistant biofilm architectures.

## 2. Adhesion in the Gram-Negative Bacteria *Pseudomonas aeruginosa* and *Escherichia coli*

The successful adhesion of Gram-negative bacteria to surfaces is largely dependent on the presence of cell appendages such as flagella, pili, and fimbriae [10]. The presence of functional flagella enables the bacterium to swim and overcome repulsive electrostatic forces that may exist between the cell surface and the surface of material or the host’s conditioning film [11]. In both *Pseudomonas aeruginosa* and *Escherichia coli* the flagellum-associated hook protein 1 is encoded by the *flgK* gene with a 40% correlation between the nucleotide sequences of the two species [12]. The processes of adhesion and accumulation in both species are outlined below.

### 2.1. Pseudomonas aeruginosa Adhesion and Accumulation

In *Pseudomonas aeruginosa*, type IV pili aid in surface adhesion. Type IV pili are constructed from a single protein subunit, PilA, that is exported out of the cell by the secretin, PilQ, to form a polymer fimbrial strand. PilA and PilQ are derived from preplins (molecules of short peptide sequences) whose synthesis is positively controlled by the *algR* regulator [13]. The *fimU-pilVWXY1Y2E* operon codes for type IV pili prepilins that gather in the periplasmic space to be cleaved and methylated by type IV prepilin peptidase [14]. Encoded in this sequence are PilY1, PilY2, and the six minor prepilins FimT, FimU, PilV, PilW, PilX, and PilE [15]. Required for pilus biosynthesis, the minor preplins are located in the cell membrane, they are not incorporated into the pili structure and are normally associated with assembly, transport, localization, maturation, and secretion of bacterial proteins [16].

PilY1 and PilY2 are also required for the formation of pili [17]. PilY1 is a large protein located both in the membrane and as part of the pili, with involvement in fimbrial assembly. PilY2 is a small protein involved in fimbrial biosynthesis. The formation of genetic mutants that lack the necessary genes to form flagella and pili/fimbriae have been shown to be surface attachment deficient with little or no biofilm formation when compared to wild-type form, thus highlighting the importance of these bacterial appendages in the adhesion process [11,18].

In *Pseudomonas aeruginosa* type-IV pili are present to aid initial adhesion in combination with two forms of the *O*-polysaccharide chain of lipopolysaccharide, labeled A and B [19]. Makin *et al.*, utilizing *Pseudomonas aeruginosa* PAO1 discovered based on environmental factors that *Pseudomonas aeruginosa* could alter its phenotypic lipopolysaccharide composition to enhance adherence, thus favoring survival and biofilm formation on a variety of biomaterial surfaces. The production of lipopolysaccharide-A increased the hydrophobicity of the cell surface and increased adhesion to hydrophobic surfaces such as polystyrene [19]. The opposite was true of lipopolysaccharide-B with increased hydrophilicity and adhesion to hydrophilic glass observed. After initial adhesion, a monolayer of *Pseudomonas aeruginosa* forms at the material surface. Movement of bacteria across the surface continues via twitching motility carried out by extension and contraction type IV pili [20]. The importance of type IV pili in biofilm architecture is demonstrated by the formation of a capped portion in the mushroom-shaped structures synonymous with *Pseudomonas aeruginosa* biofilms. These occur due to type IV pili-linked bacterial migration [21].

Intercellular adhesion of *Pseudomonas aeruginosa* cells is increased by the production of lectins, such as PA-IL and PA-IIL (also known as LecA and LecB) synthesized in the cytoplasm of planktonic cells [22]. These two internal lectins are synthesized when the cell population cannot support itself, as in the decline phase of bacterial growth or upon subjection to environmental stress. A proportion of the total bacterial population lyses, releasing these internal lectins. These newly available lectins weakly bind to healthy, uncompromised, bacterial cells with adherence to the glycoconjugate substrata. To aid in adherence PA-IL and PA-IIL are positioned in the outer membrane of biofilm bacteria [23]. PA-IL binds preferentially to galactose whereas PA-IIL has a high affinity for monosaccharides especially fucose, thus contributing to biofilm formation [24]. In *Pseudomonas aeruginosa* these lectins are soluble, with evidence to suggest they are involved in both strengthening of established biofilms and adhesion to the airways of cystic fibrosis patients [25]. Competitive inhibition of the lectin binding site, using alternative glycans such as fucose and galactose, has been studied as a potential strategy to reduce *Pseudomonas aeruginosa* exacerbations in cystic fibrosis patients [26]. Delivered as an inhalation therapy, fucose and galactose provided promising results when utilized as monotherapy or in conjunction with intravenous antibiotics. Improved lectin binding affinity was demonstrated when glycans were attached to multivalent dendrimers, suggesting a promising role as future therapeutics [27]. *Rhl* quorum sensing pathways and the stationary phase sigma factor RpoS both directly regulate the transcription of lectin-related genes (*lecA* and *lecB*) in *Pseudomonas aeruginosa* and also serve as potential therapeutic targets in the prevention of *Pseudomonas aeruginosa* biofilm formation [28].

### 2.2. Escherichia Coli Adhesion and Accumulation

*Escherichia coli* encode for pili via transcription of the *fim* gene operon with adhesion due partly to the production of type I, type IV and P pili [29]. *Escherichia coli* possess a mannose-specific FimH receptor on the tip of their type I pili that is responsible for invasion and persistence of bacteria in target cells [30]. Mannose-specific receptors aid adhesion to host tissue surfaces such as the bladder epithelium, resulting in cystitis [31]. Evidence provided by Mobley and colleagues showed that *Escherichia coli* isolates from established long-term bacteriuria, greater than 12 weeks, expressed more type I fimbriae (92% of isolates) than those in short term infections of a duration of less than 1 week (59% of isolates) [32]. A study of P fimbriae did not demonstrate persistence in the urinary tract, however proof was provided for an increase in adherence to ureteral stents when isolates possessing P fimbriae were present [33]. These results demonstrate the importance of the bacterial isolate/strain of *Escherichia coli* in the establishment of different infections. Strains of *Escherichia coli* with type I predominate in bladder infections, with P fimbriae strains usually present in kidney infections.

**Figure 1:**
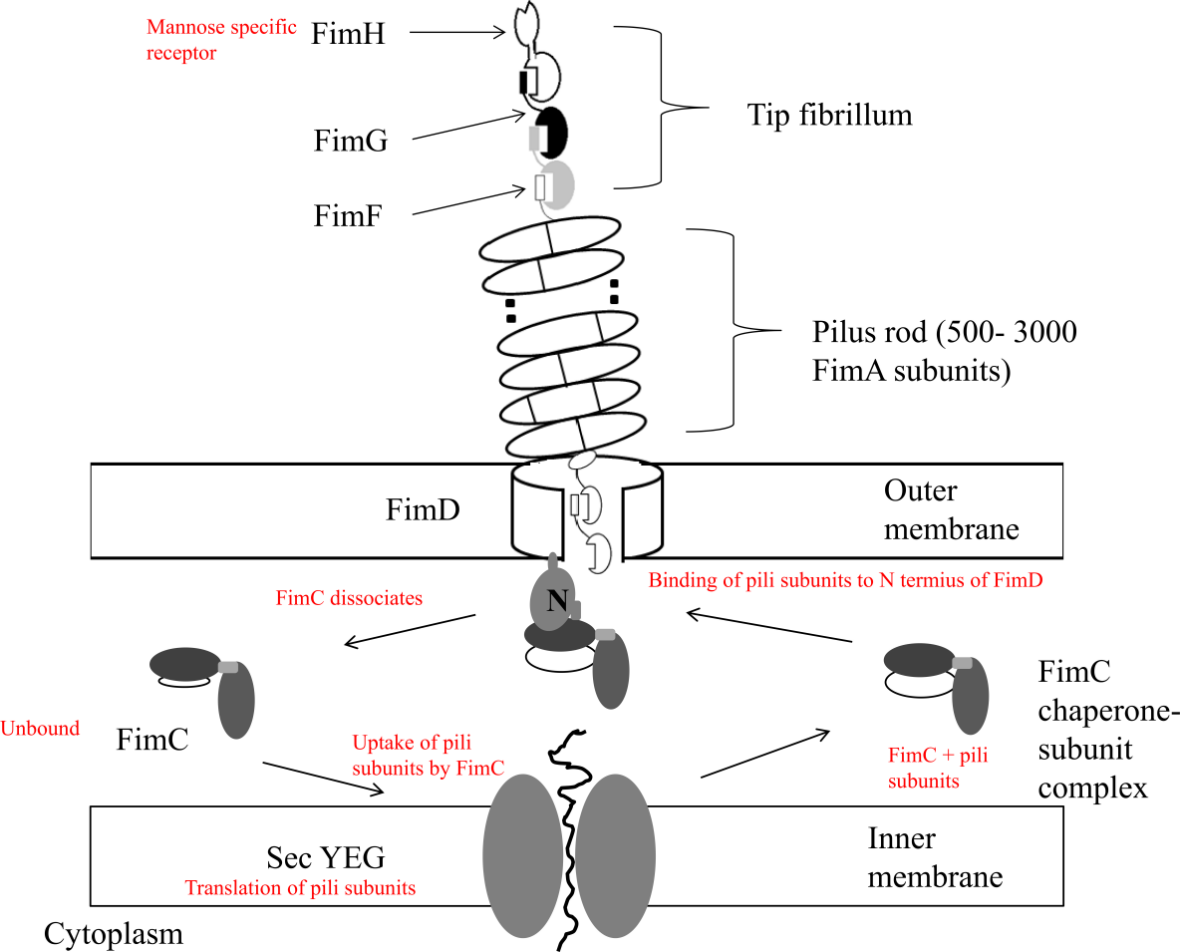
The assembly of the type I pilus. The periplasmic protein FimC binds secreted pilus subunits, from the SecYEG translocon based in the internal membrane, to the periplasm. A process of accelerated subunit folding by FimC (periplasmic chaperone) occurs, followed by delivery to the usher outer assembly platform FimD, also performed by FimC. These FimC-subunit complexes are recognized and bind to the *N*-terminal domain of the usher: FimD_N_. Uncomplexed FimC is then released to the periplasm when subunits are assembled into the pilus. The tip of the pilus (fibrillum) consists of the protein adhesins FimF, FimG, and FimH, with FimA forming the bulk of the pilus rod. Adapted from Capitani, 2006 [37].

The assembly of type I pili is controlled by the periplasmic FimC protein. FimC accelerates the folding of pilus subunits in the periplasm for delivery to the outer membrane protein FimD, where these subunits then dissociate to form the mature pilus [34] (Figure 1). FimC is termed the periplasmic chaperone and FimD the outer membrane assembly platform or usher, based on how they control type I pili synthesis [35]. FimF and FimG are linear connective proteins present in the fibrillum tip allowing the projection of the adhesin FimH that occurs only on the outer surface of the pilus [36].

**Figure 2:**
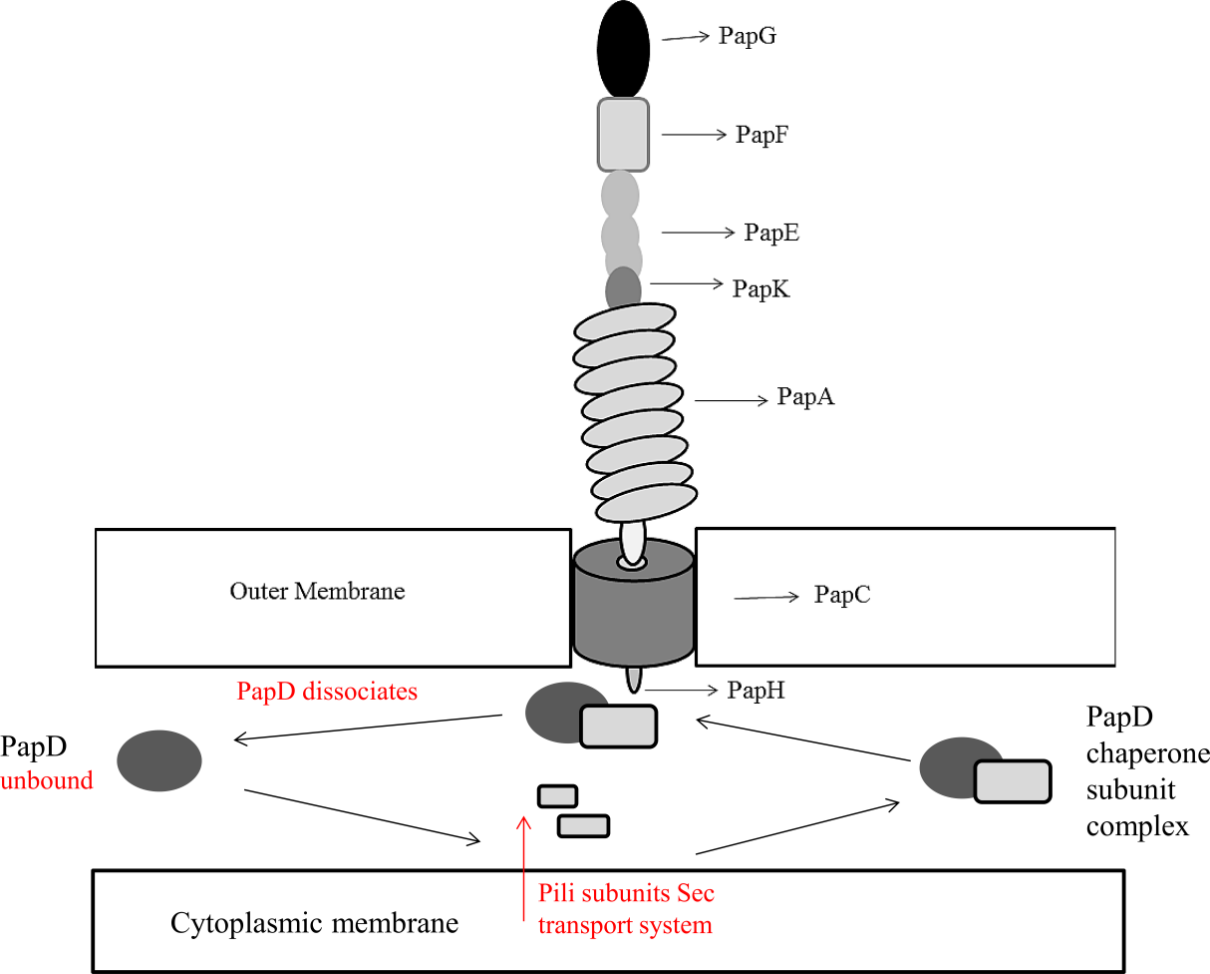
Structure of *Escherichia coli* Type P pili encompassing the PapG unit containing galabiose specific receptors (Gal(α1–4)Gal-) for attachment to urinary tract tissue. The pilus is anchored to the membrane by PapH, whose location is yet to be characterized fully but has been hypothesized by Verger and colleagues to terminate the pilus structure at the base as shown, allowing anchoring to the membrane [46]. Type P pili subunits enter the periplasm by the Sec transport system. In the presence of PapD, stable chaperone-subunit complexes are formed via attachment to the hydrophobic C-terminus of pili subunits [47]. PapD acts as the chaperone to assemble and deliver pili subunits to the outer membrane usher PapC. PapC is a pore forming protein that facilitates pilus assembly by creating a narrow channel across the outer membrane. Assembly of subunits from the outer membrane PapC occurs through a donor strand exchange mechanism. PapA forms a tightly wound helix fiber on the external cell and provides a driving force for the translocation of pili subunits across the outer membrane, facilitating outward pilus growth [48]. Adapted from Mu and Bullitt, 2006 [48] and Mu, 2005 [49].

As discussed, other forms of pili exist in *Escherichia coli* namely: P pili and type IV pili. P pili are chaperone-usher assembly mediated pili, encoded by the *pap* locus and contain galabiose specific receptors (Gal(α1–4)Gal-) on a distal PapG unit (Figure 2) [37]. This allows *Escherichia coli* to colonize the upper urinary tract, causing pyelonephritis, by binding of galabiose specific receptors (Gal(α1–4)Gal-) to the glycolipid galabiose on urinary tract tissue [38]. The fibrillum tip of P pili is composed of repeating subunits of PapE protein, with the rod consisting of PapA. PapF, PapK, and PapH proteins are also present in low quantities. PapF and PapK act as protein initiators and coordinators for assembly. PapF also acts as a linker in the fibrillum tip to PapG and PapK, thus attaching the fibrillum tip to the rod protein PapA [39]. PapH acts as the rod terminus linking it to the outer membrane surface [40]. The protein PapD acts as the periplasmic chaperone in a similar manner to FimC in type I pili [41] with PapC, like FimD, acting as the outer membrane usher [42]. Type IV pili in *Escherichia coli* are formed independently of a chaperone-usher system and are coded for by the *bfp* operon [43]. Also termed bundle-forming pili, their properties are associated with swarming and twitching motility, unlike type I and P pili, as well as adhesion [44]. Relative to type I and P pili, the formation of type IV pili is less characterized. Ramer and colleagues discovered that a *bfp* encoded assembly complex spans the entire periplasmic space and associated proteins, such as BfpU and BfpL, are present at both the inner and outer membranes [45]. They observed that a type IV related assembly complex consisted of an inner membrane component composed of three pilin-like proteins, BfpI, BfpJ and BfpK. These proteins were localized with BfpE, BfpL, and BfpA forming the major pilin subunit. BfpI, BfpJ, and BfpK were also associated with an outer membrane, secretin-like component, BfpB and BfpG, and a periplasmic component composed of BfpU. Together they create the bundle-forming pilus.

Curli fibers are organelles associated with the early stages of *Escherichia coli* adhesion and virulence. They consist of proteinaceous adhesive filaments that form a coil-like structure on the surface of *Salmonella* and *Escherichia coli*. They have an affinity for proteins such as fibronectin and are responsible for cell to cell adhesion [50]. The production of curli fibers is regulated by transcription of the *csgD* gene. CsgD protein is derived from the LuxR family of transcriptional regulators and is the activator and transcription regulator of the *csgBAC* gene operon. It is at the *csgBAC* gene loci that the protein subunits that form curli fibers are encoded [51]. CsgD also controls cellulose production, through *adrA* gene transcription, which itself is linked to the formation of an extracellular matrix [52]. Control of curli production is a very complex process with two separate gene loci required for effective curli synthesis and multiple regulatory pathways controlling their expression [53,54]. The *csgDEFG* operon encodes both the transcription of CsgD and also a curli-specific transport system mediated by CsgEFG proteins. The curli structural subunits, encoded by upregulation of the *csgBAC* gene locus, are produced in the presence of cellular and environmental stress such as low temperature (<32 °C), lack of nutrients, low osmolarity, and iron shock [55,56,57]. Limited expression of curli related genes, such as the *csgA* gene, correlates to reduced biofilm formation due to a lowering in production of the main curlin protein subunit CsgA [58]. Their importance as therapeutic targets is demonstrated by the work of Cegelski and colleagues [59]. They produced a series of ring-fused 2-pyridone containing peptidomimetic molecules (FN075 and BibC6), which prevented macromolecular assembly of the major curli subunit protein CsgA and inhibited *Escherichia coli* curli biogenesis. This resulted in reduced *Escherichia coli* colonization in the bladder of *in vivo* mouse models. These so-called curlicides also prevented type I pilus biogenesis via blockage of the FimC chaperone. Delivery of such molecules at therapeutically relevant concentrations remains a challenge that prohibits their clinical development. Changes in environmental stresses affect biofilm formation in *Escherichia coli* via the two-component regulatory system CpxA/CpxR. The CpxA/CpxR system negatively controls the transcription of the *csg*, *pap*, *bfp,* and *flgM* (*flg* are involved in flagella protein transcription and motility together with *fli*) operons [60]. CpxA is a histidine kinase involved in the transfer of a phosphate group to the regulatory protein CpxR, allowing it to bind specifically to sequences of bacterial DNA that regulate gene transcription [61]. The CpxA/CpxR system senses changes in the environmental surroundings of the periplasm, outer membrane, and bacterial envelope. Activation occurs at low nutrient concentrations, high osmolarity and high temperatures due to their effects on lipopolysaccharide and exopolysaccharide biosynthesis and the outer membrane structure [62,63]. Transcription of *CpxA*/*CpxR* system genes is controlled by the general stress response factor RpoS (stationary phase sigma factor) [64]. RpoS, also known as the alternative subunit of RNA polymerase (σ^S^), is a protein encoded by the *rpoS* gene that controls the overall response of *Escherichia coli* to environmental stress, with a sharp increase in concentrations shown at the onset of the stationary phase of growth [65]. Negative regulation of curli occurs by binding of phosphorylated CpxR to the *csgD* promoter therefore switching *csgD* expression off. In mature biofilm cells a majority of CpxA/CpxR are activated [66]. The events of initial adhesion have already occurred; therefore many of the adhesion-related appendages are not required. CpxA/CpxR expression correlates to an upregulation of genes corresponding to resistance pathways, such as the *mdtA* gene, responsible for the efflux and resistance against many β-lactam antibiotics [67]. Therefore the positive role of CpxA/CpxR is more likely to be associated with dormant or persister *Escherichia coli* cells. CpxA/CpxR is unlikely to be associated with the dispersal of biofilm cells, to facilitate recolonization of new surfaces, as genes related to motility such as flagella-related genes (*flgM*) have also been shown to be downregulated by the CpxA/CpxR system [68].

Positive regulation of curli fiber production is controlled by the EnvZ/OmpR two-component regulatory system. The OmpR protein binds to the same promoter region of *csgD* as CpxR but it is still not fully established whether they actively compete for this binding site [69]. EnvZ is a histidine kinase that controls the phosphorylation and binding-affinity of OmpR to CsgD, with phosphorylation in the presence of environmental stimuli such as high osmolarity [70]. CpxA plays a similar activating role with CpxR via a process of phosphorylation [71]. Most recently Ogasawara and colleagues analyzed mRNA of mutant *Escherichia coli* and *csgD* to indicate that CpxR and H-NS acted as repressor molecules with OmpR, an acid-stress response regulator termed RstA and IHF acting as activators within a five component system. They concluded these five factors bonded to the same narrow gene operon region of approximately 200 base pairs, upstream from the *csgD* promoter [72]. Despite the promising results obtained, the biomolecular and transcription mechanism of the *csgD* operon has not been fully elucidated. Their work showed the presence of competitive positive and negative factors but also cooperation between the positive and negative factor groups. Regulation of the *csg* loci is also controlled by the global regulatory gene *hns* [73]. The gene regulator *hns* has an established negative effect on adhesion due to upregulation of genes responsible for flagella synthesis, in comparison to *ompR,* the conclusive positive regulator of curli production [69,74].

## 3. Biofilm Maturation in *Pseudomonas Aeruginosa* and *Escherichia Coli*

Accumulation and ultimately maturation of the biofilm corresponds to the increased production of the major extracellular polymeric substance alginate in *Pseudomonas aeruginosa* [75] and colanic acid in *Escherichia coli* [76]. These compounds are important in forming the respective biofilm architecture of these microorganisms but they are not essential for biofilm formation to occur. Both species exhibit similar three-dimensional structures possessing water channels; micro and macrocolonies of significant heterogeneity and a thick biofilm matrix. Both microorganisms display downregulation of genes required for motility apparatus, specifically flagella-related genes, and upregulation in genes for extracellular polymeric substance production in the maturation stage of growth [77]. Bacterial maturation in both these Gram-negative bacteria is tightly controlled by quorum sensing systems involving *N*-acyl-l-homoserine lactone as signaling molecules, together with long-chain hydrocarbon structures derived from fatty acids, fatty acid methyl esters, peptides, γ-butyrolactones, 2-alkyl-4-quinolones, furanones, and the 4,5-dihydroxy-2,3-pentandione derivatives, collectively referred to as autoinducer-II and autoinducer-III [78,79,80,81,82].

### 3.1. Pseudomonas aeruginosa Biofilm Maturation: Production of Exopolysaccharides via the Polysaccharide Synthesis Locus (Psl), Pellicle Formation (Pel), and Alginic Acid Synthesis

The extracellular polymeric substance of *Pseudomonas aeruginosa* biofilm, in line with the majority of bacterial biofilms, consists mainly of polysaccharide, proteins, and nucleic acids [83,84,85]. In mucoid strains of *Pseudomonas aeruginosa*, isolated from cystic fibrosis patients, the most prevalent exopolysaccharide produced is alginic acid, an *O*-acetylated linear polymer of β-1,4-linked d-mannuronic acid with a C-5 epimer, l-guluronic acid [86]. Interestingly non-mucoid strains have been shown to contain low levels of alginate, with biofilm formation retained [87]. Only 1% of strains isolated from sites other than the lungs of cystic fibrosis patients are mucoid [88], therefore in relation to medical device related infection, alginic acid is not necessarily the most common exopolysaccharide present.

#### 3.1.1. Production of the Psl and Pel Exopolysaccharides by Non-Mucoid *Pseudomonas aeruginosa*

Adherence, aggregation, maturation, and formation of the biofilm architecture are also due to production the exopolysaccharides Psl and Pel. The proteins, enzymes, and transporter molecules required for Psl and Pel synthesis and pellicle formation (thin biofilm surrounding cells that assembles at the air-liquid interface) are encoded by the genes *pslA-O* and *pelA-G,* respectively, in *Pseudomonas aeruginosa* PAO1 [89]. Upon analysis of PelA-G proteins it was observed that PelA is a cytosolic protein and an oligogalacturonide lyase; Pel B functions as an outer membrane protein; PelC is a glycosyltransferase present in the periplasm; both PelD and PelE are large cytosolic proteins located on the inner membrane, with PelD an inner membrane located transmembrane protein; PelF is a glycosyltransferases and PelG is a 12-transmembrane inner membrane protein [90].

Psl proteins are not as well defined in the literature as Pel in terms of individual functions [91]. PslA was identified as a putative UDP-glucose carrier protein essential to biofilm formation in strains of *Pseudomonas aeruginosa* such as PAO1 [92]. Observations of the extracellular polymeric substances present in *Pseudomonas aeruginosa* PAO1 show that the main carbohydrate constituents are glucose, mannose, and rhamnose and not the alginic acid components mannuronate or guluronate [93]. Psl is rich in sugars, particularly mannose, with glucose, galactose, rhamnose, and a limited quantity of xylose also present [91]. The gene locus *pslA-G* is present in some strains, for example *Pseudomonas aeruginosa* PAO1, but not PA14 strains [94,95]. Pel is a glucose-rich polymer and although the genes encoding its production (*pel*) have been shown to be present in all identified strains of *Pseudomonas aeruginosa*, their expression is limited in laboratory conditions [94]. Psl is located mainly in the peripheral regions of the biofilm matrix and may have a role in attracting free-flowing planktonic bacteria to form part of the biofilm structure [84,96]. The reason for this peripheral localization is, as yet, unproven but an increase in nutrients; metabolism; DNA and protein synthesis at the outer extremities of the biofilm and/or a breakdown of Psl in the center of the matrix by the production of enzymes may contribute to this observation [97]. An interesting study by DiGiandomenico and co-workers highlighted the potential of monoclonal antibodies in combating exopolysaccharides such as Psl [98]. By performing phenotypic screening they discovered that Psl was an antibody-accessible antigen that allowed targeted monoclonal antibody mediated opsonophagocytic killing of *Pseudomonas aeruginosa*. Reduced bacterial attachment was shown with cultured lung epithelial cells and prophylactic protection provided in infected animal models. Use of such techniques may have potential for future prophylaxis against *Pseudomonas aeruginosa* infections in high-risk patients.

The production of the secondary messenger molecule bis-(3',5')-cyclic-dimeric-guanosine monophosphate (c-di-GMP) is linked to the maturation of biofilms and production of exopolysaccharides in many species of bacteria including *Pseudomonas aeruginosa*. Its production is regulated by the action of diguanylate cyclase enzymes. Cleavage of, or a decrease in, c-di-GMP production is linked to the expression of motility factors and virulence, with phosphodiesterases also linked to c-di-GMP degradation [99,100]. High levels of c-di-GMP are associated with an increase in biofilm-related traits (attachment and accumulation) [101]. A c-di-GMP binding site has also been identified on the cytosolic inner membrane protein PelD, therefore linking this molecule to Pel synthesis in *Pseudomonas aeruginosa* [90]. There has been an increasing interest in targeting c-di-GMP, or more specifically the proteins that are involved in the biosynthesis of c-di-GMP, in order to prevent biofilm formation [102]. C-di-GMP is produced from two guanosine triphosphate molecules and its synthesis is controlled by the enzyme diguanylate cyclase [103]. Irie and colleagues recently discovered that although Psl formation is controlled by c-di-GMP, it also acts as a positive feedback signal and stimulates the production of two diguanylate cyclases, SiaD and SadC, resulting in increased formation of c-di-GMP [104]. Eukaryotes do not express diguanylate cyclase, therefore it serves as an excellent target for antibacterial drug development [105]. Analogues of c-di-GMP, for example the monophosphorothioic acid of c-di-GMP (c-GpGps), display antibiofilm activity against *Pseudomonas aeruginosa* and *Staphylococcus aureus in vitro* [106]. Biofilm dispersal and an increased sensitivity to antimicrobials have also been attributed to low concentrations of nitric oxide in *Pseudomonas aeruginosa*. Barraud *et al.* uncovered a possible molecular link between nitrous oxide, reduced levels of c-di-GMP and biofilm dispersion due to an increase in phosphodiesterase activity [107]. C-di-GMP also has potential as a vaccine molecule due to its immunostimulatory and adjuvant properties [108]. The translation of such therapies to clinical practice is limited at present, as the role of c-di-GMP in biofilm formation has not been fully established. Concerns may also exist with regard to the affect such therapies may have on commensal microorganisms.

Extracellular DNA also plays an important role in biofilm maturation and stabilization of non-mucoid *Pseudomonas aeruginosa* strains such as PAO1, compensating for a lack of alginate [109]. Matsukawa *et al.* showed that in the matrix of mature *Pseudomonas aeruginosa,* PAO1 extracellular DNA was the most prevalent polymer and that exopolysaccharides were of great importance with regard to structural integrity [85]. Whitchurch and colleagues demonstrated that DNase could dissolve young *Pseudomonas aeruginosa* PAO1 biofilms, but matured biofilms showed only small dissolution. This suggests that early biofilms are held together by extracellular DNA, but mature PAO1 biofilms are held together by other compounds, namely exopolysaccharides [110]. Research by Ma demonstrated extracellular DNA to be present mostly in the stalk region of mature biofilm colonies, and spatially separate from Psl proteins [111]. Differences in the prevalence of extracellular DNA in *in vitro* and *in vivo* biofilms may be attributed to the stage of biofilm growth. Extracellular DNA may also play a role in increasing resistance of biofilm forms of *Pseudomonas aeruginosa* toward cationic antimicrobials, such as antimicrobial peptides. Extracellular DNA is a cation chelator and acts to sequester cations from the surrounding environment. It also plays a role in the modification of the cationic antimicrobial peptide binding site lipid A by the sugar dehydrogenases enzyme UDP-glucose dehydrogenase (Ugd) and covalent binding to 4-amino-4-deoxy-l-arabinose [112].

#### 3.1.2. Production of the Exopolysaccharide Alginic Acid by Mucoid *Pseudomonas aeruginosa*

Slime production by mucoid forming strains of *Pseudomonas aeruginosa* is important for the colonization of both medical devices and cell surfaces, such as the lungs in cystic fibrosis patients [113]. The formation of this slime, composed of mainly alginic acid, is important in protecting *Pseudomonas aeruginosa* from antimicrobials and host defense mechanisms by restricted penetration of these molecules through the biofilm matrix [114]. Synthesis of alginic acid, commonly known as alginate, is controlled by the *algACD* operon present in *Pseudomonas aeruginosa*. Upregulation of alginate-related genes is dependent on multiple environmental factors including: high oxygen concentration, high osmolarity, lack of nitrogen, and the presence of ethanol [115,116]. In general the production of alginic acid by the *algACD* gene locus is similar to the regulation of the *icaADBC* operon, responsible for polysaccharide intercellular adhesin production in staphylococci.

**Figure 3:**
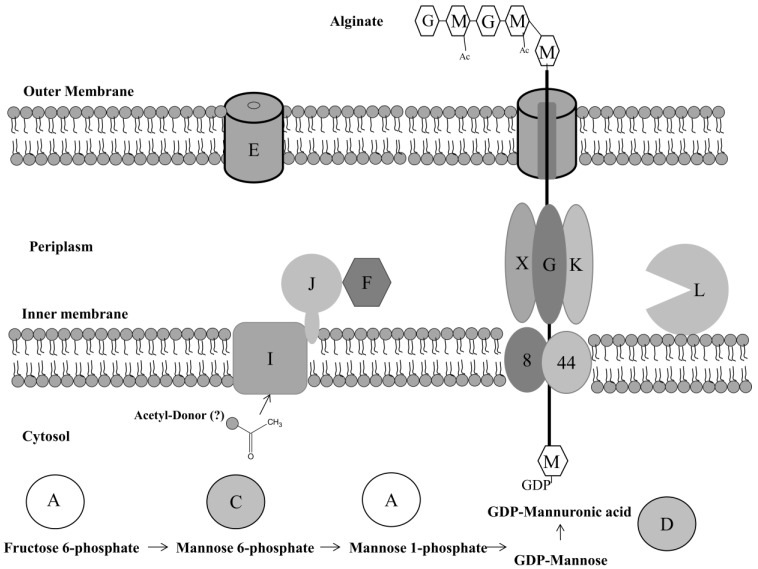
The synthesis and polymerization mechanism involved in the production of *Pseudomonas aeruginosa* alginate. The letters A–X and numbers 8 and 44 correlate to alginate biosynthetic enzymes that are preceded by Alg (for example A = AlgA). AlgA, AlgC, and AlgD control the production of the alginate precursor GDP-mannuronic acid. Both Alg8 and Alg44 transport this molecule for polymerization in the periplasm. Alginate lysase (AlgL) produces short oligomers that prime polymerization. AlgG interacts with AlgK and AlgX protecting the production of the alginate polymer by forming a scaffold in the periplasm. Epimerization of polymerized mannuronate residues is also controlled by AlgG, a C-5-epimerase. Acetylation of some mannuronate residues occurs via the enzymes AlgF, AlgJ, and AlgI at O2 and/or O3 positions, with AlgE transporting the formed alginate out of the cell. Adapted from Franklin and Ohman 2002 [122], Ramsey 2005 [125], and Gimmestad, 2003 [126].

Of high importance to alginic acid production are the *algA*, *algC,* and *algD* genes that transcribe the enzymes required for the production of the alginate precursor guanosine diphosphate (GDP)-mannuronic acid [117]. A combination of the transmembrane transporter proteins Alg44 and Alg8, which are normally not active in non-mucoid *Pseudomonas aeruginosa*, allows the movement of this alginate precursor across the inner membrane for polymerization [118]. AlgA-X are alginate enzymes involved in the polymerization and biosynthesis processes that result in the formation of alginate (Figure 3). The role of alginate lyase, at the maturation stage, is unclear although it may allow the production of short oligomers that prime polymerization and may also allow the breakdown of alginate at the cell detachment phase of biofilm growth [119]. AlgG interacts with AlgK and AlgX. They have an important role in protecting the production of the alginate polymer by forming a scaffold in the periplasm surrounding newly formed polymer molecules [120]. Epimerization of polymerized mannuronate residues is controlled by AlgG, a C-5-epimerase enzyme [121]. Acetylation of these mannuronate residues also occurs via the enzymes AlgF, AlgJ, and AlgI at O2 and/or O3 positions [122]. AlgF is located in the periplasm. AlgJ is a type II membrane protein with an uncleaved signal peptide portion linked to the inner membrane with a remaining portion in the periplasm, whereas AlgI is an integral transmembrane helix that accepts an acetyl group form an unknown donor [123]. When the process of *O*-acetylation is concluded, transportation of alginate out of the cell is mediated by AlgE present on the outer membrane forming the majority of the extracellular polymeric matrix substance of mucoid producing *Pseudomonas aeruginosa* [124].

AlgA, AlgC, and AlgD are important enzymes in the production of GDP-mannuronic acid. AlgA is involved in alginate biosynthesis catalyzing both the production of mannose-6-phosphate from fructose-6-phosphate and GDP-mannose from mannose-1-phosphate, as a phosphomannose isomerase and GDP-mannose pyrophosphorylase, respectively [127]. AlgC is a phosphomannomutase and phosphoglucomutae enzyme that catalyses the reversible production of mannose-6-phosphate to mannose-1-phosphate (Figure 3). AlgC has also been shown to be important in lipopolysaccharide synthesis, with its preference for both mannose and glucose containing substrates allowing it to possess a diverse mechanism of action [128].

AlgD is a rate-limiting GDP-mannose dehydrogenase that catalyses the production of GDP-mannuronic acid from GDP-mannose [129]. Control of alginate biosynthesis and the transcription of the Alg proteins are therefore mediated by the *algD* operon, which is responsible for the final production of GDP-mannuronic acid, the foundation molecule for polymerization and alginate synthesis. A regulatory cascade consisting of an alternative sigma factor AlgT, also known as AlgU or σ^22^ and encoded by *algT* controls the transcription of *algD* [130]. Autoregulation of *algT* and upregulation of the alginate-linked gene loci *algR*, *algB*, *algZ* are also performed by σ^22^ [131]. AlgR regulates both *algC* and *algD* transcription by binding to sites upstream of their genes [132]. AlgB also activates *algD* transcription but by an undefined method that has previously been thought to be related to indirect action of the promoter region of *algD* [133]. However, Leech and colleagues observed, through DNA binding and transcriptome analysis, that AlgB bound directly to the promoter region of the *algD* operon [134]. Transcription of *algZ*, also known as *amrZ*, leads to the activation of *algD* by binding to sequences upstream of the *algD* promoter [135]. It also inhibits transcription of the gene loci *fleQ* related to the control of flagella-related genes [136]. This demonstrates the complex systems involved in the transcription of alginate-related genes, such as *algD*, with multiple pathways involved in its transcription.

The action of σ^22^ is itself controlled by the regulatory protein products of *mucABCD* transcription [137]. The inner membrane protein MucA is an anti-sigma factor that complexes to the periplasmic protein MucB. The MucA portion then directly binds to σ^22^ after *algT* transcription to negatively regulate its activity [138]. Rowen *et al.* suggested that σ^22^ associates with the periphery of the inner membrane by interacting with RNA polymerase or MucA, or by an unknown independent mechanism blocking the action of σ^22^ [139]. MucC’s role is relatively uncharacterized, however, it is hypothesized to be an inner membrane protein that may act synergistically with both MucA and MucB in the negative regulation of σ^22^ [140]. MucD is an endoprotease that negatively regulates σ^22^ by the removal of activating factors present in σ^22^ [141]. Inactivation of *mucA* or *mucB* via mutations in non-mucoid *Pseudomonas aeruginosa* strains has been shown to induce alginate synthesis leading to an overexpression in alginate-related genes. Clinical isolates from cystic fibrosis patients have also been shown to possess mutations in *mucA*, causing an exponential increase in alginate synthesis [142,143].

### 3.2. Escherichia coli Biofilm Maturation

The process of biofilm maturation in *Escherichia coli* is very similar to *Pseudomonas aeruginosa* in that genes that encode for flagella mediated motility (*fli* and *flg*) are downregulated with corresponding upregulation of genes corresponding to the exopolysaccharide colanic acid (*wca*), porin (*ompC*) production, tripeptidase T, and synthesis of a nickel and glycine betaine high-affinity transport system [144]. As previously discussed (Section 2.2.), the Cpx/CpxR two component regulatory system, controlled by the general stress response factor RpoS (stationary phase sigma factor) [64], is responsible for the upregulation of many of the genes implicated in biofilm maturation [66].

The production of the exopolysaccharide colanic acid is essential for the maturation and complex three-dimensional structure of *Escherichia coli* biofilms but not the process of initial adhesion [145]. Colanic acid consists of hexasaccharide subunits with a high prevalence of fucose and glucuronic acid [146]. The physical barrier presented by colanic acid production and the negative charge that it possesses allows *Escherichia coli* biofilms to resist large changes in osmotic stress, oxidative stress (by hydrogen peroxide), and temperature [76]. Generally colanic acid is only produced at temperatures above 30 °C and is thought to be important in capsule formation and ultimately survival of *Escherichia coli* outside of the host [147]. The gene operon *wca*, also known as *cps*, encodes for polymerase enzymes that regulate colanic acid synthesis from sugars by a pathways that has not been elucidated fully. The transcription of the *wca* genes is controlled by the *rcsABCF* gene loci, and this pathway is more fully understood. After phosphorylation of the response regulator RcsB by the sensor kinase RscC, the accessory positive regulator RcsA binds to RcsB to form the heterodimer RcsA-RscB, which activates *wca* transcription [148]. RscF is hypothesized to promote the phosphorylation of RscB by RscC [149]. The processes and signals that cause the activation of RscC are relatively unknown [147]. It has been suggested that environmental stimuli, such as osmotic shock, play a role in the upregulation of *rscC* via changes in membrane-bound protein MdoH, which is involved in the production of membrane-derived oligosaccharides [150]. RscC senses changes in these membrane-derived oligosaccharides to initiate a response [151]. RcsA is present normally in low amounts at 37 °C due to low levels of synthesis and the presence of the protease Lon, a negative regulator of *wca* transcription [152]. The minimal level of RcsA production is due transcriptional silencing by the histone-like protein H-NS, that is negated by overproduction of a small RNA molecule known as DsrA [153].

The *wca* operon consists of 19 genes with the third gene in order of transcription being the *wzc* gene. *Wzc* has been shown to encode a membrane bound autophosphorylated protein-tyrosine kinase [154]. Upstream of this *wcz* locus, the *wca* operon codes for a phosphotyrosine phosphatase (PTP), Wcz, that has the ability to specifically dephosphorylate a corresponding protein-tyrosine kinase and is otherwise defined as a BY-kinase, a newly defined group of enzymes involved in protein-tyrosine phosphorylation [155]. Although the mechanism is unclear, dephosphorylation of Wzc tends to lead to increased colonic acid production and provides a means by which exopolysaccharides are transported out of the cell [156]. Wzc has also been shown to phosphorylate the sugar dehydrogenase enzyme Uridine diphosphate (UDP)-glucose dehydrogenase, allowing it to mediate the construction of extracellular polysaccharide precursors, such as UDP-glucuronic acid.

Etk, like Wzc, is a BY-kinase of *Escherichia coli* and is involved in the production of the group IV capsule surrounding the *Escherichia coli* bacterial cell membrane [157]. Etk is coded for by the *etk* gene present on the *ymc* operon of some pathogenic strains of *Escherichia coli* [158]. The mechanism and use of the *ymc* operon itself is unknown, although it could possibly be a promoter of *etk* expression [159]. Etk is also involved in the phosphorylation of UDP-glucose dehydrogenase allowing for the production of UDP-4-amino-4-deoxy-l-arabinose, a compound that allows *Escherichia coli* to become resistant to cationic antimicrobial peptides and polymixin-B [159].

The two-component systems PhoP/PhoQ and PmrA/PmrB are induced by limitation of magnesium and calcium ions or the RcsA/RcsB/RcsC system leading to upregulation of the genes involved in UDP-4-amino-4-deoxy-l-arabinose biosynthesis. The gene loci *arn* is controlled by both the PhoP/PhoQ and PmrA/PmrB pathways. Transcription of *arn* leads to the synthesis of UDP-4-amino-4-deoxy-l-arabinose via *arn*-linked enzymes, however synthesis of UDP-4-amino-4-deoxy-l-arabinose is also due to RcsA/RcsB/RcsC or PhoP/PhoQ and PmrA/PmrB mediated transcription of *ugd*. The protein Ugd, when phosphorylated via Etk, forms UDP-Glucuronic acid from the precursor UDP-Glucose. Formation of UDP-4-amino-4-deoxy-l-arabinose is mediated by a series of *arn* encoded enzymes [160]. Meredith *et al.* hypothesized that Ugd synthesis of colonic acid (in this case defined as M-antigen) also affects the production of lipopolysaccharides in the cell membrane via the formation of a complex lipopolysaccharide glycoform termed M_LPS_ [161]. Resistance develops against cationic antimicrobial peptides and polymyxin due to covalent modifications of lipid A, the hydrophobic anchor of the lipopolysaccharide membrane and the cationic antimicrobial binding site, by UDP-4-amino-4-deoxy-l-arabinose [160].

## 4. Quorum Sensing in Gram-Negative Bacteria: *Pseudomonas aeruginosa* and *Escherichia coli*

Quorum sensing is a system whereby bacterial cells communicate in order to act as a community of cells. This maximizes the potential of their mutualistic survival strategies, allowing selective benefits to be conferred to the bacterial population that would otherwise not be present as individual cells. Quorum sensing is of great importance in the production of bacterial biofilms and the up and down regulation of related genes [7].

### 4.1. Quorum Sensing in Pseudomonas aeruginosa

In *Pseudomonas aeruginosa* there are currently three identified quorum sensing systems. These are; the *las*-based system controlled via *N*-(3-oxododecanoyl)-l-homoserine lactone production by *lasRI* gene loci [162]; the *rhl* system regulated by *N*-butyryl-homoserine lactone produced by the *rhlRI* operon [163] and the *pqs* system that controls the *las* and *rhl* quorum sensing through 2-heptyl-3-hydroxy-4-quinolone production [164]. The *rhl* system is controlled by the *las* system as LasR-*N*-(3-oxododecanoyl)-l-homoserine lactone activates the transcription of *rhlR* and *rhlI* meaning these two systems are interlinked [165].

As for all microorganisms that demonstrate quorum sensing pathways, these signals allow for the control of phenotypic expressions such as virulence, biofilm formation, and resistance to antimicrobials [166]. In *Pseudomonas aeruginosa* quorum sensing controls the production of exoenzymes and secreted toxins, such as elastase and exotoxin A [162,167,168], and also directs biofilm formation [169]. Cells lacking in the *las* system have been shown to be flat rather than the atypical mushroom-like shape of mature *Pseudomonas aeruginosa* biofilms, with increased sensitivity to antibiotics also demonstrated [168]. It is hypothesized that the transcription of the exopolysaccharide-related *pelA-G* gene loci is controlled indirectly by *las* and *rhl* quorum sensing systems through transcriptional factors; however this process is as yet largely undefined [170]. Quorum sensing has also been shown to regulate the production of extracellular DNA in *Pseudomonas aeruginosa* biofilms [83]. The most defined quorum sensing pathways in *Pseudomonas aeruginosa* are the *las* and *rhl* systems.

The *las* system comprises of the transcriptional activator protein LasR and the autoinducer synthase enzyme LasI, coded for by *lasR* and *lasI,* respectively. The transcription of *lasI* results in the formation of the enzyme LasI that directs the synthesis of *N*-(3-oxododecanoyl)-l-homoserine lactone, an important acyl-homoserine lactone of *Pseudomonas aeruginosa*, otherwise known as autoinducer-III [171]. Increased cell density of *Pseudomonas aeruginosa* correlates to an increase in autoinducer-III concentration and when a threshold value is reached the binding of autoinducer-III to its specific target protein LasR will result in the transcription of multiple virulent and biofilm-related genes mediated by *las*. These genes include those responsible for the production of multiple enzymes such as exotoxin A (*toxA*), elastase (*lasB*), the LasA protease (*lasA*), and alkaline protease (*aprA*) [172]. The binding of autoinducer-III to LasR also results in further transcription of *lasI*, thus proving *N*-(3-oxododecanoyl)-l-homoserine lactone to be an autoinducing peptide [173]. The activation of the *las* system also results in activation of the *rhl* quorum sensing system, resulting in the production of a second autoinducer *N*-butyryl-homoserine lactone. It is therefore believed, through this mechanism, that the *las* system controls regulatory protein (RhlR) production both before and after transcription [163,173].

The *rhl* system consists of the transcriptional activator protein RhlR and the synthase RhlI that regulate *N*-butyryl-homoserine lactone production [165]. The *rhl* pathway is responsible for the production of amphiphilic biosurfactants, known as rhamnolipids (heat stable haemolysin), in the latter stages of biofilm development that aid in the maintenance of macrocolonies and fluid-filled channels [174]. The *rhl* system is also responsible for the production of multiple extracellular enzymes along with secondary metabolites such as pyocyanin, hydrogen cyanide and pyoverdin [175]. In *Pseudomonas aeruginosa* there is a high correlation between biofilm architecture and quorum sensing, with little or no influence on biofilm adhesion and motility [176].

**Figure 4:**
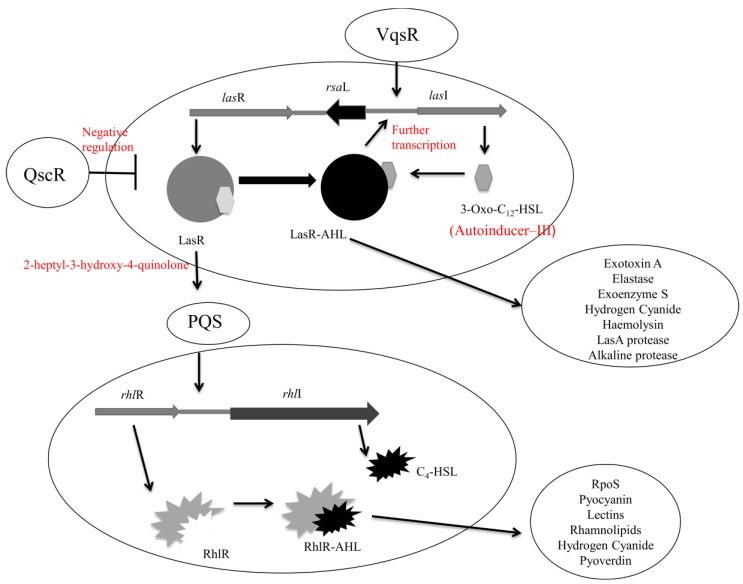
The *las*, *pqs,* and *rhl* interlinked quorum sensing systems in *Pseudomonas aeruginosa*. The *las* system consists of the proteins LasR (transcriptional activator) and LasI (synthase enzyme) coded for by *lasR* and *lasI* respectively. The *rhl* system consists of RhlR (transcriptional activator) and RhlI (synthase enzyme) coded for by *rhlR* and *rhlI*. Adapted from Raina, 2009 [177].

The third quorum sensing system identified in *Pseudomonas aeruginosa* is the *pqs* system that utilizes 2-heptyl-3-hydroxy-4-quinolone, also known as *Pseudomonas* quinolone signal. The *pqs* system has particular importance for the production of *rhl*-dependent exoproducts at the beginning of the stationary phase of growth [177]. The production of 2-heptyl-3-hydroxy-4-quinolone is related to the activation of *lasR* and is therefore thought to operate between the *las* and *rhl* systems (Figure 4) [178]. The 2-heptyl-3-hydroxy-4-quinolone molecule differs from that of acyl-homoserine lactone in that it is capable of overcoming cell density dependent production of exoproducts but not growth-phase dependent production [179]. *Pseudomonas aeruginosa* mutants defective at producing 2-heptyl-3-hydroxy-4-quinolone have lower levels of exoproducts but similar levels of the lectin adhesin PA-IL as the wild type [180]. By supplying exogenous 2-heptyl-3-hydroxy-4-quinolone exoproduct levels are restored to wild type [181]. The similar levels of lectin adhesin (PA-IL) present in mutant defective *Pseudomonas aeruginosa* suggest its control via *lecA* transcription may be due in part to an alternative system, namely the stationary phase sigma factor RpoS [28].

The *las* system is regulated directly by a variety of complex factors. Two LuxR homologues, known as quorum sensing control repressor (QscR) and virulence quorum sensing regulator (VqsR), are responsible for the negative and positive regulation of the *las* system, respectively, by unquantified mechanisms [182]. It is hypothesized that QscR negatively regulates *las* by repressing *lasI* transcription in the lag phase of growth when the concentration of autoinducing signal molecules has not reached its threshold for quorum sensing activation. QscR is thought to achieve repression by forming heterodimers of both LasR and RglR that are inactive but compete with acyl-homoserine lactones for binding to their relative cognate response regulator up to the threshold concentration. Above the threshold concentration these heterodimers dissociate allowing the formation of active LasR and RhlR homodimers [183].

VqsR allows positive regulation of quorum sensing pathways. Mutant strains of *Pseudomonas aeruginosa*, with no *vqsR* gene, show a lack of acyl-homoserine lactone production corresponding to a reduction in virulence and pathogenicity by an as yet unestablished mechanism [182]. The complexity of quorum sensing control is shown by the multitude of factors controlling its regulation. Rsal is a transcriptional regulator that acts to block *lasI* transcription by competitively binding to LasI and thus preventing LasR-*N*-(3-oxododecanoyl)-l-homoserine lactone complexes from autoinducing LasI production [172]. This is likely to occur below threshold concentrations of *N*-(3-oxododecanoyl)-l-homoserine lactone in response to an uncharacterized environmental or metabolic stimulus [184,185]. There are a variety of accounts in the literature were synthetic derivatives of *N*-l-homoserine lactones result in inhibition of quorum sensing pathways and reduced biofilm development. Of particular relevance is the study by Hentzer and colleagues [186]. They demonstrated that synthesized compounds based on natural furanones were shown to be potent antagonists of *las* and *rhl* quorum sensing in *Pseudomonas aeruginosa*. Similarly Geske *et al.* synthesized small molecular weight, non-native *N*-l-homoserine lactone derivatives that blocked natural signals via binding to LasR in *Pseudomonas aeruginosa* [187].

### 4.2. RpoS and Quorum Sensing

RpoS, also known as the alternative subunit of RNA polymerase or the stationary phase sigma factor, is a protein encoded by the *rpoS* gene and controls the overall response of both *Pseudomonas aeruginosa* and *Escherichia coli* to environmental stress. A sharp increase in RpoS concentration has been demonstrated at the onset of the stationary phase of growth [75]. In *Pseudomonas aeruginosa*, mutation of *rpoS* leads to enhanced susceptibility of stationary-phase cells to heat, high osmolarity, acidic (low) pH, hydrogen peroxide, and ethanol [188]. The stationary phase sigma factor RpoS is also controlled directly by the *rhl* system and therefore indirectly by *las*, enabling the quorum sensing control of genes at the stationary phase [165,188,189,190]. Their interaction is complex, a relationship has been observed but no obvious mechanism apparent. Whiteley *et al.* suggest RpoS is responsible for negatively regulating *rhlI* transcription, especially in hydrogen cyanide (*hcnABC*) and phenazine (*phzABC*) genes [190]. The production of the lectin adhesin PA-IL and possibly PA-IL, encoded by *lecA* and *lecB,* respectively, are mediated both directly by the *rhl*-dependent regulation of the *lecA* and indirectly by *rhl* though RpoS [28]. Other studies, such as those by Medina *et al.*, suggest RpoS partially activate the *rhlAB* genes that transcribe rhamnolipid production with a possibility that RpoS activates quorum sensing linked genes required in the stationary phase only [191]. Schuster *et al.* proposed that RpoS acts by repressing *rhlI* at the log phase of growth thus decreasing *N*-butyryl-homoserine lactone production, with loss of this repression at the late logarithmic to stationary phase of growth [192].

### 4.3. Quorum Sensing in Escherichia coli

Quorum sensing in *Escherichia coli* occurs via four different systems; the LuxR homologue *sdiA* system [193,194]; the *luxS*/autoinducer-II system [195]; an autoinducer-III/epinephrine/norepinephrine system [78], and an indole mediated system [196].

#### 4.3.1. *sdiA* Quorum Sensing System

The *sdiA* quorum sensing system provides a means by which *Escherichia coli* can sense the autoinducer *N*-acyl-l-homoserine lactone from other species of microorganisms. SdiA is a LuxR homologue that binds to *N*-acyl-l-homoserine lactone and has links to the upregulation of the cell division gene operon *ftsQAZ* [197]. In other microorganisms, such as *Vibrio fischeri*, the LuxI protein [198] (encoded by *luxI*) allows the formation of *N*-acyl-l-homoserine lactone. The *luxI* gene is not present in *Escherichia coli* and thus *N*-acyl-l-homoserine lactone must be synthesized by other microorganisms [199]. This quorum sensing system of importance to *Escherichia coli* is present in the gut microflora, where other species of *N*-acyl-l-homoserine lactone producing bacteria are prevalent. Nature is proving to be a ubiquitous source of quorum sensing inhibitors. Research performed recently by Ravichandiran demonstrated that extracts from seeds of the Melia dubia plant contained compounds that competitively inhibited SdiA, resulting in reduced biofilm formation in *Escherichia coli* [200]. Other natural sources of quorum sensing inhibitors include garlic, marine algae, and soil bacteria [201,202,203]. Difficulties remain with regard to identification, structural elucidation, isolation, and purification of natural sourced components.

#### 4.3.2. *luxS* Quorum Sensing System

**Figure 5:**
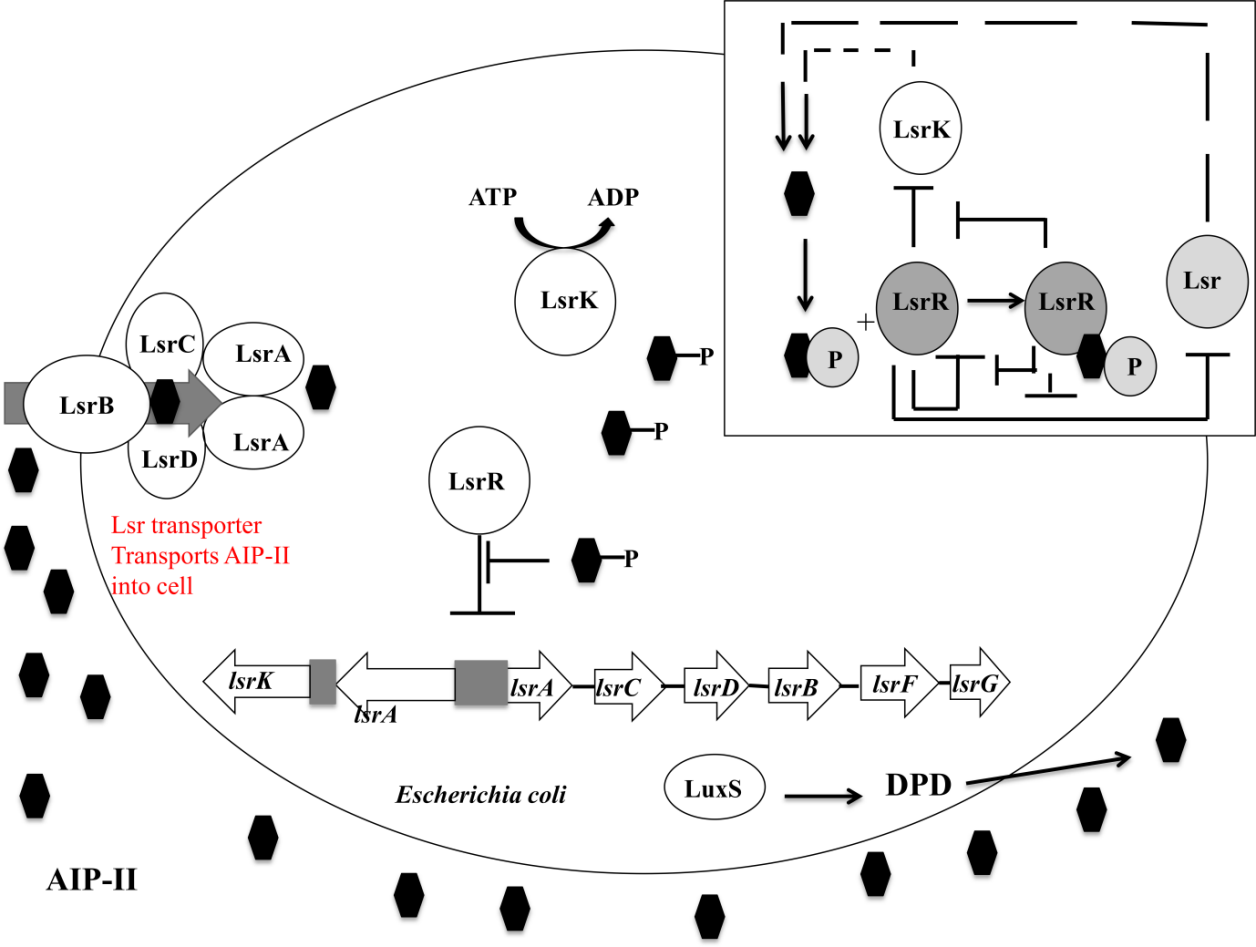
Summary of the *LuxS* Quorum sensing system of *Escherichia coli*. Autoinducer-II, represented by pentagons, is formed from a LuxS catalyzed cleavage reaction of *S*-ribosylhomocysteine to 4,5-dihydroxy 2,3-pentanedione and homocysteine [213]. Key: AIP-II: Autoinducer-II: 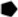 DPD: 4,5-dihydroxy-2,3-pentanedione. Adapted from Li, 2007 [184].

The *luxS* system in *Escherichia coli* has autoinducer-II as its mediating quorum-sensing molecule, and synthesis of autoinducer-II is as described for the staphylococcal *luxS* system [7]. The *luxS* system may also have a role in cell metabolism through the intracellular activated methyl cycle, together with up and downregulation of quorum sensing-related genes [80,204]. Concentration of extracellular autoinducer-II reaches a peak at the mid- to late-exponential phase with a large decrease as bacteria enter the stationary phase, but there is no relative decrease in LuxS protein levels at the stationary phase of growth [205,206]. Decrease in the concentration of extracellular autoinducer-II, at the onset of the stationary phase, corresponds to an increase in autoinducer-II uptake into cells via an ATP-binding cassette deemed the Lsr transporter that is itself *luxS*-regulated [206]. Uptake of autoinducer-II, via an alternative active transport mechanism or diffusion, may be due to the bacteria requiring autoinducer-II to regulate gene expression and therefore switch off external metabolic and cellular responses. The Lsr transporter is encoded by the *lsrABCD* operon, with LsrB required for autoinducer-II transport into the cell, and whose transcription itself is regulated via the proteins LsrK and LsrR (transcribed from *lsrRK* operon) [207]. LsrK, present in the cytoplasm, is a kinase that donates a phosphate group to autoinducer-II, allowing the phosphorylated autoinducer-II to bind to the *lsr* repressor LsrR (Figure 5). Phosphorylation may act to sequester autoinducer-II activity within the cell, although its purpose is generally undefined [193]. Mutants of *lsrK*, deficient in LsrK production, have shown the Lsr transporter to be repressed, thus autoinducer-II remains in the extracellular fluid [207]. Mutants of *lsrR* express Lsr transporter and therefore autoinducer-II is imported into the cell cytoplasm continuously [208].

LsrK and LsrR are both quorum sensing regulators. A study of how the separate deletion of each corresponded to the resulting phenotypic profile was conducted by Li *et al.* [209]. They observed that genes associated with adhesion, such as the curli-related genes CsgA, CsgE, CsgF, and CsgG, together with the pili gene *htrE*, a homolog of *papD*, were all downregulated with increased intracellular autoinducer-II in *lsrR* mutants [210,211]. In *lsrK* mutants, fimbrial genes were downregulated with two putative fimbrial proteins, *yadK* and *yadN*, repressed. The large complexity of colonic acid synthesis and *luxS* quorum sensing in general is shown by upregulation of the *wza* gene in both mutants with *wcaA* upregulation in *lsrR* mutants corresponding to an increase in intracellular autoinducer-II. The complexity involved in having extracellular and intracellular, as well as both quorum sensing and metabolic roles for autoinducer-II, is further demonstrated by the work of DeLisa *et al.* [212]. They observed that autoinducer-II upregulated a series of genes involved in fimbriae (*yadK* and *yadN*, putative fimbrial proteins), curli fiber (*crl*, the transcriptional regulator of cryptic *csgA* gene), and colonic acid production (*wzb*, a probable protein-tyrosine-phosphatase and *rscB*), with associated down regulation of genes linked to flagella synthesis (*flgN*). No link to either the intracellular or extracellular action of autoinducer-II was provided.

Further reactions form autoinducer-II. LsrR acts as the autoinducer-II uptake repressor repressing the *lsrACDBFG* and *lsrRK* operons [207]. At early and mid-exponential phases, the intracellular and extracellular autoinducer-II levels are low, thus LsrR can bind to and repress these genes [206]. As the levels of autoinducer-II increase extracellularly, active transport and diffusion of this molecule into the cell occurs by an uncharacterized non-Lsr related pathway [195]. Non-phosphorylated autoinducer-II binds to LsrR to depress many quorum sensing and biofilm forming genes including; *lsrR* itself; *flu* which is responsible for the phase-variable protein antigen 43 linked to autoaggregation; *wza* the gene linked to colonic acid synthesis; *dsrA* which encodes a small RNA molecule known as DsrA resulting in increased RcsA and upregulation of the colonic acid producing *wca* operon [153]. Lsr autoinducer-II uptake remains repressed until the late exponential phase whereby a threshold concentration of autoinducer-II is reached, corresponding to nutrient depletion, and leading to rapid autoinducer-II uptake by the Lsr transporter. Autoinducer-II is phosphorylated by LsrK (boxed section) with binding of this molecule to LsrR causing a cessation of *lsr* repression [214]. Transcription of the *lsr* operon acts as a positive feedback loop, importing more autoinducer-II in response to detection of phosphorylated autoinducer-II [215]. This leads to a relative decrease in LsrR/autoinducer-II quorum sensing and an increase in the LsrR/Phosphorylated autoinducer-II quorum sensing system, triggering the expression of biofilm linked genes.

#### 4.3.3. Autoinducer-III/Epinephrine/Norepinephrine and Indole Quorum Sensing Systems

The LuxS enzyme is also responsible for the formation of another 4,5-dihydroxy-2,3-pentandione derivative, autoinducer-III [216]. This system is present within enterohemorrhagic *Escherichia coli,* which causes bloody diarrhoea linked to haemorrhagic colitis and haemolytic-uremic syndrome [217]. The formation of autoinducer-III is not fully reliant on LuxS. A *luxS* mutation leaves the cell with only one pathway to produce homocysteine, a molecule required for autoinducer-III synthesis, and produced by other microorganisms present as part of the normal microbial gut flora [218]. Autoinducer-III is not linked directly to biofilm formation in *Escherichia coli*. Its use is mainly focused on genes related to virulence factors, adhesion in the large intestine (*LEE* operon and attaching and effacing lesions) and motility (flagella) [219]. The catecholamine class of hormones also act as agonists to this system, in particular epinephrine and norepinephrine, which are produced by the colonized host [78,220]. The autoinducer-III/epinephrine/norepinephrine quorum sensing system involves a set of regulon. The QsecC regulon autophosphorylates in the presence of both autoinducer-III and epinephrine, phosphorylating the QseB response regulator to activate the genes responsible for flagella synthesis [221]. The attaching and effacing lesions required for intestinal attachment are controlled by a similar QseE and QseF system [222]. Two LysR transcriptional factors, QseA and QseD control *LEE* transcription [223]. The indole mediated quorum sensing signal is thought to allow *Escherichia coli* to adapt to environments where nutrients are poor and the breakdown of amino acids serves as an energy source. Indole itself is formed from the breakdown of tryptophan by the tryptophanase enzyme encoded by the *tna* gene. The production of indole activates this *tna* gene and also the *astD* and *gabT* genes, which code for enzymes that control the degradation of amino acids to pyruvate or succinate [196,224].

## 5. Conclusions

The biomolecular processes that govern biofilm formation in Gram-negative *Escherichia coli* and *Pseudomonas aeruginosa* display intricate differences and are excellent examples as to the complexities that govern the survival of microbial communities. Antimicrobial resistance is increasing worldwide. This together with a relative lack of innovative antimicrobials being released to the pharmaceutical market has led to real concerns from the World Health Organization. Their 2014 global report on surveillance of antimicrobial resistance outlined the real possibility of a future whereby common infections and minor injuries can kill, including those by Gram-negative bacteria such as *Escherichia coli* [225]. Biofilm formation is one of the most important phenotypic traits in determining the resistance characteristics of microbial communities to current therapeutic strategies [226]. Therefore a comprehensive understanding of the biomolecular mechanisms allows targeted therapies to be developed in order to prevent the development of, or to eradicate, established biofilms. Thereby limiting pathogenicity in areas such as medical device related infections. O’Loughlin and colleagues recently demonstrated that small synthetically produced molecules, such as meta-bromo-thiolactone, were able to inhibit both *Pseudomonas aeruginosa* LasR and RhlR *in vitro* and *in vivo* resulting in inhibition of biofilm formation [227]. Major challenges still exist with regard to translating these molecules to produce effective therapeutic applications. One significant obstacle is that blocking a biofilm linked pathway may lead to upregulation of genes involved in the production of virulent factors [228]. Processes such as quorum sensing are often strain or isolate specific. Further research is required into control and feedback mechanisms of each biomolecular system and how they are inherently interlinked to produce phenotypic traits. Sophisticated control and modulation, rather than a policy of complete negation, of multiple pathways will be required tailored individual etiology of the infectious disease.

## References

[1] Christensen L.D., Moser C., Jensen P.O., Rasmussen T.B., Christophersen L., Kjelleberg S., Kumar N., Hoiby N., Givskov M., Bjarnsholt T. (2007). Impact of *Pseudomonas aeruginosa* Quorum Sensing on Biofilm Persistence in an *in Vivo* Intraperitoneal Foreign-Body Infection Model. Microbiology.

[2] Cole S.J., Records A.R., Orr M.W., Linden S.B., Lee V.T. (2014). Catheter-Associated Urinary Tract Infection by *Pseudomonas aeruginosa* is Mediated by Exopolysaccharide-Independent Biofilms. Infect. Immun..

[3] Guggenbichler J.P., Assadian O., Boeswald M., Kramer A. (2011). Incidence and Clinical Implication of Nosocomial Infections Associated with Implantable Biomaterials—Catheters, Ventilator-Associated Pneumonia, Urinary Tract Infections. GMS Krankenhhyg. Interdiszip..

[4] Ramos G.P., Rocha J.L., Tuon F.F. (2013). Seasonal Humidity may Influence *Pseudomonas aeruginosa* Hospital-Acquired Infection Rates. Int. J. Infect. Dis..

[5] Hoiby N., Ciofu O., Bjarnsholt T. (2010). *Pseudomonas aeruginosa* Biofilms in Cystic Fibrosis. Future Microbiol..

[6] Jacobsen S.M., Stickler D.J., Mobley H.L., Shirtliff M.E. (2008). Complicated Catheter-Associated Urinary Tract Infections due to *Escherichia coli* and *Proteus mirabilis*. Clin. Microbiol. Rev..

[7] Foxman B., Brown P. (2003). Epidemiology of Urinary Tract Infections: Transmission and Risk Factors, Incidence, and Costs. Infect. Dis. Clin. N. Am..

[8] Laverty G., Gorman S.P., Gilmore B.F. (2013). Biomolecular Mechanisms of Staphylococcal Biofilm Formation. Future Microbiol..

[9] Sutherland I.W. (2001). Microbial Polysaccharides from Gram-negative Bacteria. Int. Dairy J..

[10] Lejeune P. (2003). Contamination of Abiotic Surfaces: What a Colonizing Bacterium Sees and how to Blur it. Trends Microbiol..

[11] O’Toole G.A., Kolter R. (1998). Flagellar and Twitching Motility are Necessary for *Pseudomonas aeruginosa* Biofilm Development. Mol. Microbiol..

[12] Dunne W.M. (2002). Bacterial Adhesion: Seen any Good Biofilms Lately?. Clin. Microbiol. Rev..

[13] Bohn Y.S., Brandes G., Rakhimova E., Horatzek S., Salunkhe P., Munder A., van Barneveld A., Jordan D., Bredenbruch F., Haussler S. (2009). Multiple Roles of *Pseudomonas aeruginosa* TBCF10839 PilY1 in Motility, Transport and Infection. Mol. Microbiol..

[14] Strom M.S., Nunn D.N., Lory S. (1994). Posttranslational Processing of Type IV Prepilin and Homologs by PilD of *Pseudomonas aeruginosa*. Methods Enzymol..

[15] Mattick J.S. (2002). Type IV Pili and Twitching Motility. Ann. Rev. Microbiol..

[16] Nunn D., Bergman S., Lory S. (1990). Products of Three Accessory Genes, *pilB*, *pilC*, and *pilD*, are Required for Biogenesis of *Pseudomonas aeruginosa* Pili. J. Bacteriol..

[17] Alm R.A., Hallinan J.P., Watson A.A., Mattick J.S. (1996). Fimbrial Biogenesis Genes of *Pseudomonas aeruginosa*: *pilW* and *pilX* Increase the Similarity of Type 4 Fimbriae to the GSP Protein-Secretion Systems and *pilY1* Encodes a Gonococcal PilC Homologue. Mol. Microbiol..

[18] Murray T.S., Kazmierczak B.I. (2008). *Pseudomonas aeruginosa* Exhibits Sliding Motility in the Absence of Type IV Pili and Flagella. J. Bacteriol..

[19] Makin S.A., Beveridge T.J. (1996). The Influence of A-Band and B-Band Lipopolysaccharide on the Surface Characteristics and Adhesion of *Pseudomonas aeruginosa* to Surfaces. Microbiology.

[20] Darzins A., Russell M.A. (1997). Molecular Genetic Analysis of Type-4 Pilus Biogenesis and Twitching Motility using *Pseudomonas aeruginosa* as a Model System-a Review. Gene.

[21] Barken K.B., Pamp S.J., Yang L., Gjermansen M., Bertrand J.J., Klausen M., Givskov M., Whitchurch C.B., Engel J.N., Tolker-Nielsen T. (2008). Roles of Type IV Pili, Flagellum-Mediated Motility and Extracellular DNA in the Formation of Mature Multicellular Structures in *Pseudomonas aeruginosa* Biofilms. Environ. Microbiol..

[22] Wentworth J.S., Austin F.E., Garber N., Gilboa-Garber N., Paterson C.A., Doyle R.J. (1991). Cytoplasmic Lectins Contribute to the Adhesion of *Pseudomonas aeruginosa*. Biofouling.

[23] Tielker D., Hacker S., Loris R., Strathmann M., Wingender J., Wilhelm S., Rosenau F., Jaeger K.E. (2005). *Pseudomonas aeruginosa* Lectin LecB is Located in the Outer Membrane and is Involved in Biofilm Formation. Microbiology.

[24] Adam J., Pokorna M., Sabin C., Mitchell E.P., Imberty A., Wimmerova M. (2007). Engineering of PA-IIL Lectin from *Pseudomonas aeruginosa*—Unravelling the Role of the Specificity Loop for Sugar Preference. BMC Struct. Biol..

[25] Mewe M., Tielker D., Schonberg R., Schachner M., Jaeger K.E., Schumacher U. (2005). *Pseudomonas aeruginosa* Lectins I and II and their Interaction with Human Airway Cilia. J. Laryngol. Otol..

[26] Hauber H.P., Schulz M., Pforte A., Mack D., Zabel P., Schumacher U. (2008). Inhalation with Fucose and Galactose for Treatment of *Pseudomonas aeruginosa* in Cystic Fibrosis Patients. Int. J. Med. Sci..

[27] Kolomiets E., Swiderska M.A., Kadam R.U., Johansson E.M., Jaeger K.E., Darbre T., Reymond J.L. (2009). Glycopeptide Dendrimers with High Affinity for the Fucose-Binding Lectin LecB from *Pseudomonas aeruginosa*. ChemMedChem.

[28] Winzer K., Falconer C., Garber N.C., Diggle S.P., Camara M., Williams P. (2000). The *Pseudomonas aeruginosa* Lectins PA-IL and PA-IIL are Controlled by Quorum Sensing and by RpoS. J. Bacteriol..

[29] Hull R.A., Gill R.E., Hsu P., Minshew B.H., Falkow S. (1981). Construction and Expression of Recombinant Plasmids Encoding Type 1 Or D-Mannose-Resistant Pili from a Urinary Tract Infection *Escherichia coli* Isolate. Infect. Immun..

[30] Baorto D.M., Gao Z., Malaviya R., Dustin M.L., van der Merwe A., Lublin D.M., Abraham S.N. (1997). Survival of FimH-Expressing Enterobacteria in Macrophages Relies on Glycolipid Traffic. Nature.

[31] Connell I., Agace W., Klemm P., Schembri M., Marild S., Svanborg C. (1996). Type 1 Fimbrial Expression Enhances *Escherichia coli* Virulence for the Urinary Tract. Proc. Natl. Acad. Sci. USA.

[32] Mobley H.L., Chippendale G.R., Tenney J.H., Hull R.A., Warren J.W. (1987). Expression of Type 1 Fimbriae may be Required for Persistence of *Escherichia coli* in the Catheterized Urinary Tract. J. Clin. Microbiol..

[33] Cormio L., Vuopio-Varkila J., Siitonen A., Talja M., Ruutu M. (1996). Bacterial Adhesion and Biofilm Formation on various Double-J Stents *in Vivo* and *in Vitro*. Scand. J. Urol. Nephrol..

[34] Vetsch M., Puorger C., Spirig T., Grauschopf U., Weber-Ban E.U., Glockshuber R. (2004). Pilus Chaperones Represent a New Type of Protein-Folding Catalyst. Nature.

[35] Sauer F.G., Barnhart M., Choudhury D., Knight S.D., Waksman G., Hultgren S.J. (2000). Chaperone-Assisted Pilus Assembly and Bacterial Attachment. Curr. Opin. Struct. Biol..

[36] Gossert A.D., Bettendorff P., Puorger C., Vetsch M., Herrmann T., Glockshuber R., Wuthrich K. (2008). NMR Structure of the *Escherichia coli* Type 1 Pilus Subunit FimF and its Interactions with Other Pilus Subunits. J. Mol. Biol..

[37] Capitani G., Eidam O., Glockshuber R., Grutter M.G. (2006). Structural and Functional Insights into the Assembly of Type 1 Pili from *Escherichia coli*. Microbes Infect..

[38] Lugmaier R.A., Schedin S., Kuhner F., Benoit M. (2008). Dynamic Restacking of *Escherichia coli* P-Pili. Eur. Biophys. J..

[39] Jacob-Dubuisson F., Heuser J., Dodson K., Normark S., Hultgren S. (1993). Initiation of Assembly and Association of the Structural Elements of a Bacterial Pilus Depend on Two Specialized Tip Proteins. EMBO J..

[40] Baga M., Norgren M., Normark S. (1987). Biogenesis of *E. coli* Pap Pili: PapH, a Minor Pilin Subunit Involved in Cell Anchoring and Length Modulation. Cell.

[41] Jones C.H., Danese P.N., Pinkner J.S., Silhavy T.J., Hultgren S.J. (1997). The Chaperone-Assisted Membrane Release and Folding Pathway is Sensed by Two Signal Transduction Systems. EMBO J..

[42] Thanassi D.G., Saulino E.T., Lombardo M.J., Roth R., Heuser J., Hultgren S.J. (1998). The PapC Usher Forms an Oligomeric Channel: Implications for Pilus Biogenesis Across the Outer Membrane. Proc. Natl. Acad. Sci. USA.

[43] Anantha R.P., Stone K.D., Donnenberg M.S. (2000). Effects of *bfp* Mutations on Biogenesis of Functional Enteropathogenic *Escherichia coli* Type IV Pili. J. Bacteriol..

[44] Strom M.S., Lory S. (1993). Structure-Function and Biogenesis of the Type IV Pili. Ann. Rev. Microbiol..

[45] Ramer S.W., Schoolnik G.K., Wu C.Y., Hwang J., Schmidt S.A., Bieber D. (2002). The Type IV Pilus Assembly Complex: Biogenic Interactions among the Bundle-Forming Pilus Proteins of Enteropathogenic *Escherichia coli*. J. Bacteriol..

[46] Sauer F.G., Knight S.D., Waksman and G.J., Hultgren S.J. (2000). PapD-Like Chaperones and Pilus Biogenesis. Semin. Cell Dev. Biol..

[47] Verger D., Miller E., Remaut H., Waksman G., Hultgren S. (2006). Molecular Mechanism of P Pilus Termination in Uropathogenic *Escherichia coli*. EMBO Rep..

[48] Mu X.Q., Bullitt E. (2006). Structure and Assembly of P-Pili: A Protruding Hinge Region used for Assembly of a Bacterial Adhesion Filament. Proc. Natl. Acad. Sci. USA.

[49] Mu X.Q., Jiang Z.G., Bullitt E. (2005). Localization of a Critical Interface for Helical Rod Formation of Bacterial Adhesion P-Pili. J. Mol. Biol..

[50] Olsen A., Arnqvist A., Hammar M., Sukupolvi S., Normark S. (1993). The RpoS Sigma Factor Relieves H-NS-Mediated Transcriptional Repression of *csgA*, the Subunit Gene of Fibronectin-Binding Curli in *Escherichia coli*. Mol. Microbiol..

[51] Arnqvist A., Olsen A., Normark S. (1994). Sigma S-Dependent Growth-Phase Induction of the *csgBA* Promoter in *Escherichia coli* can be Achieved *in Vivo* by Sigma 70 in the Absence of the Nucleoid-Associated Protein H-NS. Mol. Microbiol..

[52] Romling U., Rohde M., Olsen A., Normark S., Reinkoster J. (2000). AgfD, the Checkpoint of Multicellular and Aggregative Behaviour in *Salmonella typhimurium* Regulates at Least Two Independent Pathways. Mol. Microbiol..

[53] Brown P.K., Dozois C.M., Nickerson C.A., Zuppardo A., Terlonge J., Curtiss R. (2001). MlrA, a Novel Regulator of Curli (AgF) and Extracellular Matrix Synthesis by *Escherichia coli* and *Salmonella enterica* Serovar Typhimurium. Mol. Microbiol..

[54] Gerstel U., Park C., Romling U. (2003). Complex Regulation of *csgD* Promoter Activity by Global Regulatory Proteins. Mol. Microbiol..

[55] Gerstel U., Romling U. (2001). Oxygen Tension and Nutrient Starvation are Major Signals that Regulate *agfD* Promoter Activity and Expression of the Multicellular Morphotype in *Salmonella typhimurium*. Environ. Microbiol..

[56] Olsen A., Arnqvist A., Hammar M., Normark S. (1993). Environmental Regulation of Curli Production in *Escherichia coli*. Infect. Agents Dis..

[57] Romling U., Sierralta W.D., Eriksson K., Normark S. (1998). Multicellular and Aggregative Behaviour of *Salmonella typhimurium* Strains is Controlled by Mutations in the *agfD* Promoter. Mol. Microbiol..

[58] Dorel C., Vidal O., Prigent-Combaret C., Vallet I., Lejeune P. (1999). Involvement of the Cpx Signal Transduction Pathway of *E. coli* in Biofilm Formation. FEMS Microbiol. Lett..

[59] Cegelski L., Pinkner J.S., Hammer N.D., Cusumano C.K., Hung C.S., Chorell E., Aberg V., Walker J.N., Seed P.C., Almqvist F. (2009). Small-Molecule Inhibitors Target *Escherichia coli* Amyloid Biogenesis and Biofilm Formation. Nat. Chem. Biol..

[60] Dorel C., Lejeune P., Rodrigue A. (2006). The Cpx System of *Escherichia coli*, a Strategic Signaling Pathway for Confronting Adverse Conditions and for Settling Biofilm Communities?. Res. Microbiol..

[61] Landini P. (2009). Cross-Talk Mechanisms in Biofilm Formation and Responses to Environmental and Physiological Stress in *Escherichia coli*. Res. Microbiol..

[62] Danese P.N., Oliver G.R., Barr K., Bowman G.D., Rick P.D., Silhavy T.J. (1998). Accumulation of the Enterobacterial Common Antigen Lipid II Biosynthetic Intermediate Stimulates *degP* Transcription in *Escherichia coli*. J. Bacteriol..

[63] Prigent-Combaret C., Brombacher E., Vidal O., Ambert A., Lejeune P., Landini P., Dorel C. (2001). Complex Regulatory Network Controls Initial Adhesion and Biofilm Formation in *Escherichia coli* Via Regulation of the *csgD* Gene. J. Bacteriol..

[64] De Wulf P., Kwon O., Lin E.C. (1999). The CpxRA Signal Transduction System of *Escherichia coli*: Growth-Related Autoactivation and Control of Unanticipated Target Operons. J. Bacteriol..

[65] Becker G., Klauck E., Hengge-Aronis R. (1999). Regulation of RpoS Proteolysis in *Escherichia coli*: The Response Regulator RssB is a Recognition Factor that Interacts with the Turnover Element in RpoS. Proc. Natl. Acad. Sci. USA.

[66] Beloin C., Valle J., Latour-Lambert P., Faure P., Kzreminski M., Balestrino D., Haagensen J.A., Molin S., Prensier G., Arbeille B., Ghigo J.M. (2004). Global Impact of Mature Biofilm Lifestyle on *Escherichia coli* K-12 Gene Expression. Mol. Microbiol..

[67] Hirakawa H., Inazumi Y., Masaki T., Hirata T., Yamaguchi A. (2005). Indole Induces the Expression of Multidrug Exporter Genes in *Escherichia coli*. Mol. Microbiol..

[68] De Wulf P., McGuire A.M., Liu X., Lin E.C. (2002). Genome-Wide Profiling of Promoter Recognition by the Two-Component Response Regulator CpxR-P in *Escherichia coli*. J. Biol. Chem..

[69] Jubelin G., Vianney A., Beloin C., Ghigo J.M., Lazzaroni J.C., Lejeune P., Dorel C. (2005). CpxR/OmpR Interplay Regulates Curli Gene Expression in Response to Osmolarity in *Escherichia coli*. J. Bacteriol..

[70] Rampersaud A., Harlocker S.L., Inouye M. (1994). The OmpR Protein of *Escherichia coli* Binds to Sites in the *ompF* Promoter Region in a Hierarchical Manner Determined by its Degree of Phosphorylation. J. Biol. Chem..

[71] Yamamoto K., Hirao K., Oshima T., Aiba H., Utsumi R., Ishihama A. (2005). Functional Characterization *in Vitro* of all Two-Component Signal Transduction Systems from *Escherichia coli*. J. Biol. Chem..

[72] Ogasawara H., Yamada K., Kori A., Yamamoto K., Ishihama A. (2010). Regulation of the *Escherichia coli*
*csgD* Promoter: Interplay between Five Transcription Factors. Microbiology.

[73] Vidal O., Longin R., Prigent-Combaret C., Dorel C., Hooreman M., Lejeune P. (1998). Isolation of an *Escherichia coli* K-12 Mutant Strain Able to Form Biofilms on Inert Surfaces: Involvement of a New *ompR* Allele that Increases Curli Expression. J. Bacteriol..

[74] Landini P., Zehnder A.J. (2002). The Global Regulatory *hns* Gene Negatively Affects Adhesion to Solid Surfaces by Anaerobically Grown *Escherichia coli* by Modulating Expression of Flagellar Genes and Lipopolysaccharide Production. J. Bacteriol..

[75] Evans L.R., Linker A. (1973). Production and Characterization of the Slime Polysaccharide of *Pseudomonas aeruginosa*. J. Bacteriol..

[76] Chen J., Lee S.M., Mao Y. (2004). Protective Effect of Exopolysaccharide Colanic Acid of *Escherichia coli* O157:H7 to Osmotic and Oxidative Stress. Int. J. Food Microbiol..

[77] Zhao K., Liu M., Burgess R.R. (2007). Adaptation in Bacterial Flagellar and Motility Systems: From Regulon Members to Foraging-Like Behavior in *E. coli*. Nucl. Acids Res..

[78] Sperandio V., Torres A.G., Jarvis B., Nataro J.P., Kaper J.B. (2003). Bacteria-Host Communication: The Language of Hormones. Proc. Natl. Acad. Sci. USA.

[79] Winzer K., Williams P. (2003). *Escherichia coli* Gets the Message. Nat. Med..

[80] Vendeville A., Winzer K., Heurlier K., Tang C.M., Hardie K.R. (2005). Making “Sense” of Metabolism: Autoinducer-2, LuxS and Pathogenic Bacteria. Nat. Rev. Microbiol..

[81] Williams P., Winzer K., Chan W.C., Camara M. (2007). Look Who’s Talking: Communication and Quorum Sensing in the Bacterial World. Philos. Trans. R. Soc. Lond. Ser. B Biol. Sci..

[82] Winzer K., Hardie K.R., Williams P. (2002). Bacterial Cell-to-Cell Communication: Sorry, can’t Talk Now—Gone to Lunch!. Curr. Opin. Microbiol..

[83] Allesen-Holm M., Barken K.B., Yang L., Klausen M., Webb J.S., Kjelleberg S., Molin S., Givskov M., Tolker-Nielsen T.A. (2006). Characterization of DNA Release in *Pseudomonas aeruginosa* Cultures and Biofilms. Mol. Microbiol..

[84] Branda S.S., Vik S., Friedman L., Kolter R. (2005). Biofilms: The Matrix Revisited. Trends Microbiol..

[85] Matsukawa M., Greenberg E.P. (2004). Putative Exopolysaccharide Synthesis Genes Influence *Pseudomonas aeruginosa* Biofilm Development. J. Bacteriol..

[86] Deretic V., Govan J.R., Konyecsni W.M., Martin D.W. (1990). Mucoid *Pseudomonas aeruginosa* in Cystic Fibrosis: Mutations in the *muc* Loci Affect Transcription of the *algR* and *algD* Genes in Response to Environmental Stimuli. Mol. Microbiol..

[87] Stapper A.P., Narasimhan G., Ohman D.E., Barakat J., Hentzer M., Molin S., Kharazmi A., Hoiby N., Mathee K. (2004). Alginate Production Affects *Pseudomonas aeruginosa* Biofilm Development and Architecture, but is Not Essential for Biofilm Formation. J. Med. Microbiol..

[88] Doggett R.G., Harrison G.M., Stillwell R.N., Wallis E.S. (1966). An Atypical *Pseudomonas aeruginosa* Associated with Cystic Fibrosis of the Pancreas. J. Pediatr..

[89] Friedman L., Kolter R. (2004). Two Genetic Loci Produce Distinct Carbohydrate-Rich Structural Components of the *Pseudomonas aeruginosa* Biofilm Matrix. J. Bacteriol..

[90] Lee V.T., Matewish J.M., Kessler J.L., Hyodo M., Hayakawa Y., Lory S. (2007). A Cyclic-Di-GMP Receptor Required for Bacterial Exopolysaccharide Production. Mol. Microbiol..

[91] Byrd M.S., Sadovskaya I., Vinogradov E., Lu H., Sprinkle A.B., Richardson S.H., Ma L., Ralston B., Parsek M.R., Anderson E.M. (2009). Genetic and Biochemical Analyses of the *Pseudomonas aeruginosa* Psl Exopolysaccharide Reveal Overlapping Roles for Polysaccharide Synthesis Enzymes in Psl and LPS Production. Mol. Microbiol..

[92] Overhage J., Schemionek M., Webb J.S., Rehm B.H. (2005). Expression of the *psl* Operon in *Pseudomonas aeruginosa* PAO1 Biofilms: PslA Performs an Essential Function in Biofilm Formation. Appl. Environ. Microbiol..

[93] Wozniak D.J., Wyckoff T.J., Starkey M., Keyser R., Azadi P., O’Toole G.A., Parsek M.R. (2003). Alginate is Not a Significant Component of the Extracellular Polysaccharide Matrix of PA14 and PAO1 *Pseudomonas aeruginosa* Biofilms. Proc. Natl. Acad. Sci. USA.

[94] Friedman L., Kolter R. (2004). Genes Involved in Matrix Formation in *Pseudomonas aeruginosa* PA14 Biofilms. Mol. Microbiol..

[95] Stewart P.S., Franklin M.J. (2008). Physiological Heterogeneity in Biofilms. Nat. Rev. Microbiol..

[96] Ma L., Lu H., Sprinkle A., Parsek M.R., Wozniak D.J. (2007). *Pseudomonas aeruginosa* Psl is a Galactose- and Mannose-Rich Exopolysaccharide. J. Bacteriol..

[97] Rani S.A., Pitts B., Beyenal H., Veluchamy R.A., Lewandowski Z., Davison W.M., Buckingham-Meyer K., Stewart P.S. (2007). Spatial Patterns of DNA Replication, Protein Synthesis, and Oxygen Concentration within Bacterial Biofilms Reveal Diverse Physiological States. J. Bacteriol..

[98] DiGiandomenico A., Warrener P., Hamilton M., Guillard S., Ravn P., Minter R., Camara M.M., Venkatraman V., Macgill R.S., Lin J. (2012). Identification of Broadly Protective Human Antibodies to *Pseudomonas aeruginosa* Exopolysaccharide Psl by Phenotypic Screening. J. Exp. Med..

[99] Christen B., Christen M., Paul R., Schmid F., Folcher M., Jenoe P., Meuwly M., Jenal U. (2006). Allosteric Control of Cyclic Di-GMP Signaling. J. Biol. Chem..

[100] Kulasakara H., Lee V., Brencic A., Liberati N., Urbach J., Miyata S., Lee D.G., Neely A.N., Hyodo M., Hayakawa Y. (2006). Analysis of *Pseudomonas aeruginosa* Diguanylate Cyclases and Phosphodiesterases Reveals a Role for Bis-(3'-5')-Cyclic-GMP in Virulence. Proc. Natl. Acad. Sci. USA.

[101] Belas R. (2014). Biofilms, flagella, and mechanosensing of surfaces by bacteria. Trends Microbiol..

[102] Sintim H.O., Smith J.A., Wang J., Nakayama S., Yan L. (2010). Paradigm Shift in Discovering Next-Generation Anti-Infective Agents: Targeting Quorum Sensing, c-Di-GMP Signaling and Biofilm Formation in Bacteria with Small Molecules. Future Med. Chem..

[103] Castiglione N., Stelitano V., Rinaldo S., Giardina G., Caruso M., Cutruzzolà F. (2011). Metabolism of Cyclic-Di-GMP in Bacterial Biofilms: From a General Overview to Biotechnological Applications. Indian J. Biotechnol..

[104] Irie Y., Borlee B.R., O’Connor J.R., Hill P.J., Harwood C.S., Wozniak D.J., Parsek M.R. (2012). Self-Produced Exopolysaccharide is a Signal that Stimulates Biofilm Formation in *Pseudomonas aeruginosa*. Proc. Natl. Acad. Sci. USA.

[105] Anantharaman V., Iyer L.M., Aravind L. (2010). Presence of a Classical RRM-Fold Palm Domain in Thg1-Type 3'- 5'Nucleic Acid Polymerases and the Origin of the GGDEF and CRISPR Polymerase Domains. Biol. Direct.

[106] Mano E., Hyodo M., Sato Y., Ishihara Y., Ohta M., Hayakawa Y. (2007). Synthesis of Cyclic Bis(3'-5')-2'-deoxyguanylic/guanylic Acid (c-dGpGp) and its Biological Activities to Microbes. ChemMedChem.

[107] Barraud N., Schleheck D., Klebensberger J., Webb J.S., Hassett D.J., Rice S.A., Kjelleberg S. (2009). Nitric Oxide Signaling in *Pseudomonas aeruginosa* Biofilms Mediates Phosphodiesterase Activity, Decreased Cyclic Di-GMP Levels, and Enhanced Dispersal. J. Bacteriol..

[108] Yan H., Wang X., KuoLee R., Chen W. (2008). Synthesis and Immunostimulatory Properties of the Phosphorothioate Analogues of cdiGMP. Bioorg. Med. Chem. Lett..

[109] Ghafoor A., Hay I.D., Rehm B.H. (2011). Role of Exopolysaccharides in *Pseudomonas aeruginosa* Biofilm Formation and Architecture. Appl. Environ. Microbiol..

[110] Whitchurch C.B., Tolker-Nielsen T., Ragas P.C., Mattick J.S. (2002). Extracellular DNA Required for Bacterial Biofilm Formation. Science.

[111] Ma L., Jackson K.D., Landry R.M., Parsek M.R., Wozniak D.J. (2006). Analysis of *Pseudomonas aeruginosa* Conditional Psl Variants Reveals Roles for the Psl Polysaccharide in Adhesion and Maintaining Biofilm Structure Postattachment. J. Bacteriol..

[112] Mulcahy H., Charron-Mazenod L., Lewenza S. (2008). Extracellular DNA Chelates Cations and Induces Antibiotic Resistance in *Pseudomonas aeruginosa* Biofilms. PLoS Pathog..

[113] May T.B., Shinabarger D., Maharaj R., Kato J., Chu L., DeVault J.D., Roychoudhury S., Zielinski N.A., Berry A., Rothmel R.K. (1991). Alginate Synthesis by *Pseudomonas aeruginosa*: A Key Pathogenic Factor in Chronic Pulmonary Infections of Cystic Fibrosis Patients. Clin. Microbiol. Rev..

[114] Kumon H., Tomochika K., Matunaga T., Ogawa M., Ohmori H. (1994). A Sandwich Cup Method for the Penetration Assay of Antimicrobial Agents through *Pseudomonas* Exopolysaccharides. Microbiol. Immunol..

[115] DeVault J.D., Kimbara K., Chakrabarty A.M. (1990). Pulmonary Dehydration and Infection in Cystic Fibrosis: Evidence that Ethanol Activates Alginate Gene Expression and Induction of Mucoidy in *Pseudomonas aeruginosa*. Mol. Microbiol..

[116] Zielinski N.A., Maharaj R., Roychoudhury S., Danganan C.E., Hendrickson W., Chakrabarty A.M. (1992). Alginate Synthesis in *Pseudomonas aeruginosa*: Environmental Regulation of the *algC* Promoter. J. Bacteriol..

[117] Gacesa P. (1998). Bacterial Alginate Biosynthesis-Recent Progress and Future Prospects. Microbiology.

[118] Maharaj R., May T.B., Wang S.K., Chakrabarty A.M. (1993). Sequence of the *alg*8 and *alg*44 Genes Involved in the Synthesis of Alginate by *Pseudomonas aeruginosa*. Gene.

[119] Boyd A., Chakrabarty A.M. (1994). Role of Alginate Lyase in Cell Detachment of *Pseudomonas aeruginosa*. Appl. Environ. Microbiol..

[120] Robles-Price A., Wong T.Y., Sletta H., Valla S., Schiller N.L. (2004). AlgX is a Periplasmic Protein Required for Alginate Biosynthesis in *Pseudomonas aeruginosa*. J. Bacteriol..

[121] Douthit S.A., Dlakic M., Ohman D.E., Franklin M.J. (2005). Epimerase Active Domain of *Pseudomonas aeruginosa* AlgG, a Protein that Contains a Right-Handed Beta-Helix. J. Bacteriol..

[122] Franklin M.J., Ohman D.E. (2002). Mutant Analysis and Cellular Localization of the AlgI, AlgJ, and AlgF Proteins Required for O Acetylation of Alginate in *Pseudomonas aeruginosa*. J. Bacteriol..

[123] Franklin M.J., Douthit S.A., McClure M.A. (2004). Evidence that the *algI*/*algJ* Gene Cassette, Required for O Acetylation of *Pseudomonas aeruginosa* Alginate, Evolved by Lateral Gene Transfer. J. Bacteriol..

[124] Rehm B.H., Valla S. (1997). Bacterial Alginates: Biosynthesis and Applications. Appl. Microbiol. Biotechnol..

[125] Ramsey D.M., Wozniak D.J. (2005). Understanding the Control of *Pseudomonas aeruginosa* Alginate Synthesis and the Prospects for Management of Chronic Infections in Cystic Fibrosis. Mol. Microbiol..

[126] Gimmestad M., Sletta H., Ertesvag H., Bakkevig K., Jain S., Suh S.J., Skjak-Braek G., Ellingsen T.E., Ohman D.E., Valla S. (2003). The *Pseudomonas* Fluorescens AlgG Protein, but Not its Mannuronan C-5-Epimerase Activity, is Needed for Alginate Polymer Formation. J. Bacteriol..

[127] Shinabarger D., Berry A., May T.B., Rothmel R., Fialho A., Chakrabarty A.M. (1991). Purification and Characterization of Phosphomannose Isomerase-Guanosine Diphospho-d-Mannose Pyrophosphorylase. A Bifunctional Enzyme in the Alginate Biosynthetic Pathway of *Pseudomonas aeruginosa*. J. Biol. Chem..

[128] Regni C., Naught L., Tipton P.A., Beamer L.J. (2004). Structural Basis of Diverse Substrate Recognition by the Enzyme PMM/PGM from *P. aeruginosa*. Structure.

[129] Govan J.R., Deretic V. (1996). Microbial Pathogenesis in Cystic Fibrosis: Mucoid *Pseudomonas aeruginosa* and *Burkholderia. cepacia*. Microbiol. Rev..

[130] DeVries C.A., Ohman D.E. (1994). Mucoid-to-Nonmucoid Conversion in Alginate-Producing *Pseudomonas aeruginosa* often Results from Spontaneous Mutations in *algT*, Encoding a Putative Alternate Sigma Factor, and shows Evidence for Autoregulation. J. Bacteriol..

[131] Wozniak D.J., Sprinkle A.B., Baynham P.J. (2003). Control of *Pseudomonas aeruginosa*
*algZ* Expression by the Alternative Sigma Factor AlgT. J. Bacteriol..

[132] Nikolskaya A.N., Galperin M.Y. (2002). A Novel Type of Conserved DNA-Binding Domain in the Transcriptional Regulators of the AlgR/AgrA/LytR Family. Nucl. Acids Res..

[133] Woolwine S.C., Wozniak D.J. (1999). Identification of an *Escherichia coli*
*pepA* Homolog and its Involvement in Suppression of the *algB* Phenotype in Mucoid *Pseudomonas aeruginosa*. J. Bacteriol..

[134] Leech A.J., Sprinkle A., Wood L., Wozniak D.J., Ohman D.E. (2008). The NtrC Family Regulator AlgB, which Controls Alginate Biosynthesis in Mucoid *Pseudomonas aeruginosa*, Binds Directly to the *algD* Promoter. J. Bacteriol..

[135] Baynham P.J., Wozniak D.J. (1996). Identification and Characterization of AlgZ, an AlgT-Dependent DNA-Binding Protein Required for *Pseudomonas aeruginosa*
*algD* Transcription. Mol. Microbiol..

[136] Tart A.H., Blanks M.J., Wozniak D.J. (2006). The AlgT-Dependent Transcriptional Regulator AmrZ (AlgZ) Inhibits Flagellum Biosynthesis in Mucoid, Nonmotile *Pseudomonas aeruginosa* Cystic Fibrosis Isolates. J. Bacteriol..

[137] Mathee K., McPherson C.J., Ohman D.E. (1997). Posttranslational Control of the *algT* (*algU*)-Encoded sigma22 for Expression of the Alginate Regulon in *Pseudomonas aeruginosa* and Localization of its Antagonist Proteins MucA and MucB (AlgN). J. Bacteriol..

[138] Schurr M.J., Yu H., Martinez-Salazar J.M., Boucher J.C., Deretic V. (1996). Control of AlgU, a Member of the Sigma E-Like Family of Stress Sigma Factors, by the Negative Regulators MucA and MucB and *Pseudomonas aeruginosa* Conversion to Mucoidy in Cystic Fibrosis. J. Bacteriol..

[139] Rowen D.W., Deretic V. (2000). Membrane-to-Cytosol Redistribution of ECF Sigma Factor AlgU and Conversion to Mucoidy in *Pseudomonas aeruginosa* Isolates from Cystic Fibrosis Patients. Mol. Microbiol..

[140] Boucher J.C., Schurr M.J., Yu H., Rowen D.W., Deretic V. (1997). *Pseudomonas aeruginosa* in Cystic Fibrosis: Role of *mucC* in the Regulation of Alginate Production and Stress Sensitivity. Microbiology.

[141] Wood L.F., Ohman D.E. (2006). Independent Regulation of MucD, an HtrA-Like Protease in *Pseudomonas aeruginosa*, and the Role of its Proteolytic Motif in Alginate Gene Regulation. J. Bacteriol..

[142] Goldberg J.B., Gorman W.L., Flynn J.L., Ohman D.E. (1993). A Mutation in *algN* Permits Trans Activation of Alginate Production by *algT* in *Pseudomonas* Species. J. Bacteriol..

[143] Martin D.W., Schurr M.J., Mudd M.H., Deretic V. (1993). Differentiation of *Pseudomonas aeruginosa* into the Alginate-Producing Form: Inactivation of *mucB* Causes Conversion to Mucoidy. Mol. Microbiol..

[144] Prigent-Combaret C., Vidal O., Dorel C., Lejeune P. (1999). Abiotic Surface Sensing and Biofilm-Dependent Regulation of Gene Expression in *Escherichia coli*. J. Bacteriol..

[145] Danese P.N., Pratt L.A., Kolter R. (2000). Exopolysaccharide Production is Required for Development of *Escherichia coli* K12 Biofilm Architecture. J. Bacteriol..

[146] Obadia B., Lacour S., Doublet P., Baubichon-Cortay H., Cozzone A.J., Grangeasse C. (2007). Influence of Tyrosine-Kinase Wzc Activity on Colanic Acid Production in *Escherichia coli* K12 Cells. J. Mol. Biol..

[147] Whitfield C., Roberts I.S. (1999). Structure, Assembly and Regulation of Expression of Capsules in *Escherichia coli*. Mol. Microbiol..

[148] Ebel W., Trempy J.E. (1999). *Escherichia coli* RcsA, a Positive Activator of Colanic Acid Capsular Polysaccharide Synthesis, Functions to Activate its Own Expression. J. Bacteriol..

[149] Gervais F.G., Drapeau G.R. (1992). Identification, Cloning, and Characterization of *rcsF*, a New Regulator Gene for Exopolysaccharide Synthesis that Suppresses the Division Mutation ftsZ84 in *Escherichia coli* K-12. J. Bacteriol..

[150] Lacroix J.M., Loubens I., Tempete M., Menichi B., Bohin J.P. (1991). The *mdoA* Locus of *Escherichia coli* Consists of an Operon Under Osmotic Control. Mol. Microbiol..

[151] Ebel W., Vaughn G.J., Peters H.K., Trempy J.E. (1997). Inactivation of *mdoH* Leads to Increased Expression of Colanic Acid Capsular Polysaccharide in *Escherichia coli*. J. Bacteriol..

[152] Sailer F.C., Meberg B.M., Young K.D. (2003). Beta-Lactam Induction of Colanic Acid Gene Expression in *Escherichia coli*. FEMS Microbiol. Lett..

[153] Sledjeski D., Gottesman S. (1995). A Small RNA Acts as an Antisilencer of the H-NS-Silenced *rcsA* Gene of *Escherichia coli*. Proc. Natl. Acad. Sci. USA.

[154] Vincent C., Doublet P., Grangeasse C., Vaganay E., Cozzone A.J., Duclos B. (1999). Cells of *Escherichia coli* Contain a Protein-Tyrosine Kinase, Wzc, and a Phosphotyrosine-Protein Phosphatase, Wzb. J. Bacteriol..

[155] Grangeasse C., Cozzone A.J., Deutscher J., Mijakovic I. (2007). Tyrosine Phosphorylation: An Emerging Regulatory Device of Bacterial Physiology. Trends Biochem. Sci..

[156] Mijakovic I., Poncet S., Boel G., Maze A., Gillet S., Jamet E., Decottignies P., Grangeasse C., Doublet P., le Marechal P., Deutscher J. (2003). Transmembrane Modulator-Dependent Bacterial Tyrosine Kinase Activates UDP-Glucose Dehydrogenases. EMBO J..

[157] Lee D.C., Zheng J., She Y.M., Jia Z. (2008). Structure of *Escherichia coli* Tyrosine Kinase Etk Reveals a Novel Activation Mechanism. EMBO J..

[158] Peleg A., Shifrin Y., Ilan O., Nadler-Yona C., Nov S., Koby S., Baruch K., Altuvia S., Elgrably-Weiss M., Abe C.M., Knutton S., Saper M.A., Rosenshine I. (2005). Identification of an *Escherichia coli* Operon Required for Formation of the *O*-Antigen Capsule. J. Bacteriol..

[159] Lacour S., Bechet E., Cozzone A.J., Mijakovic I., Grangeasse C. (2008). Tyrosine Phosphorylation of the UDP-Glucose Dehydrogenase of *Escherichia coli* is at the Crossroads of Colanic Acid Synthesis and Polymyxin Resistance. PLoS One.

[160] Raetz C.R., Reynolds C.M., Trent M.S., Bishop R.E. (2007). Lipid A Modification Systems in Gram-negative Bacteria. Ann. Rev. Biochem..

[161] Meredith T.C., Mamat U., Kaczynski Z., Lindner B., Holst O., Woodard R.W. (2007). Modification of Lipopolysaccharide with Colanic Acid (M-Antigen) Repeats in *Escherichia coli*. J. Biol. Chem..

[162] Passador L., Cook J.M., Gambello M.J., Rust L., Iglewski B.H. (1993). Expression of *Pseudomonas aeruginosa* Virulence Genes Requires Cell-to-Cell Communication. Science.

[163] Ochsner U.A., Reiser J. (1995). Autoinducer-Mediated Regulation of Rhamnolipid Biosurfactant Synthesis in *Pseudomonas aeruginosa*. Proc. Natl. Acad. Sci. USA.

[164] Diggle S.P., Cornelis P., Williams P., Camara M. (2006). 4-Quinolone Signalling in *Pseudomonas aeruginosa*: Old Molecules, New Perspectives. Int. J. Med. Microbiol..

[165] Latifi A., Foglino M., Tanaka K., Williams P., Lazdunski A. (1996). A Hierarchical Quorum-Sensing Cascade in *Pseudomonas aeruginosa* Links the Transcriptional Activators LasR and RhIR (VsmR) to Expression of the Stationary-Phase Sigma Factor RpoS. Mol. Microbiol..

[166] Wagner V.E., Bushnell D., Passador L., Brooks A.I., Iglewski B.H. (2003). Microarray Analysis of *Pseudomonas aeruginosa* Quorum-Sensing Regulons: Effects of Growth Phase and Environment. J. Bacteriol..

[167] Winson M.K., Camara M., Latifi A., Foglino M., Chhabra S.R., Daykin M., Bally M., Chapon V., Salmond G.P., Bycroft B.W. (1995). Multiple *N*-Acyl-l-Homoserine Lactone Signal Molecules Regulate Production of Virulence Determinants and Secondary Metabolites in *Pseudomonas aeruginosa*. Proc. Natl. Acad. Sci. USA.

[168] Davies D.G., Parsek M.R., Pearson J.P., Iglewski B.H., Costerton J.W., Greenberg E.P. (1998). The Involvement of Cell-to-Cell Signals in the Development of a Bacterial Biofilm. Science.

[169] Hentzer M., Eberl L., Nielsen J., Givskov M. (2003). Quorum Sensing: A Novel Target for the Treatment of Biofilm Infections. BioDrugs.

[170] Sakuragi Y., Kolter R. (2007). Quorum-Sensing Regulation of the Biofilm Matrix Genes (*pel*) of *Pseudomonas aeruginosa*. J. Bacteriol..

[171] Pearson J.P., Gray K.M., Passador L., Tucker K.D., Eberhard A., Iglewski B.H., Greenberg E.P. (1994). Structure of the Autoinducer Required for Expression of *Pseudomonas aeruginosa* Virulence Genes. Proc. Natl. Acad. Sci. USA.

[172] De Kievit T.R., Iglewski B.H. (2000). Bacterial Quorum Sensing in Pathogenic Relationships. Infect. Immun..

[173] Pesci E.C., Iglewski B.H. (1997). The Chain of Command in *Pseudomonas* Quorum Sensing. Trends Microbiol..

[174] Davey M.E., Caiazza N.C., O’Toole G.A. (2003). Rhamnolipid Surfactant Production Affects Biofilm Architecture in *Pseudomonas aeruginosa* PAO1. J. Bacteriol..

[175] Williams P., Camara M. (2009). Quorum Sensing and Environmental Adaptation in *Pseudomonas aeruginosa*: A Tale of Regulatory Networks and Multifunctional Signal Molecules. Curr. Opin. Microbiol..

[176] Beatson S.A., Whitchurch C.B., Semmler A.B., Mattick J.S. (2002). Quorum Sensing is Not Required for Twitching Motility in *Pseudomonas aeruginosa*. J. Bacteriol..

[177] Raina S., de Vizio D., Odell M., Clements M., Vanhulle S., Keshavarz T. (2009). Microbial Quorum Sensing: A Tool Or a Target for Antimicrobial Therapy?. Biotechnol. Appl. Biochem..

[178] McKnight S.L., Iglewski B.H., Pesci E.C. (2000). The *Pseudomonas* Quinolone Signal Regulates *rhl* Quorum Sensing in *Pseudomonas aeruginosa*. J. Bacteriol..

[179] Diggle S.P., Winzer K., Chhabra S.R., Worrall K.E., Camara M., Williams P. (2003). The *Pseudomonas aeruginosa* Quinolone Signal Molecule Overcomes the Cell Density-Dependency of the Quorum Sensing Hierarchy, Regulates *rhl*-Dependent Genes at the Onset of Stationary Phase and can be Produced in the Absence of LasR. Mol. Microbiol..

[180] Pesci E.C., Milbank J.B., Pearson J.P., McKnight S., Kende A.S., Greenberg E.P., Iglewski B.H. (1999). Quinolone Signaling in the Cell-to-Cell Communication System of *Pseudomonas aeruginosa*. Proc. Natl. Acad. Sci. USA.

[181] Aendekerk S., Diggle S.P., Song Z., Hoiby N., Cornelis P., Williams P., Camara M. (2005). The MexGHI-OpmD Multidrug Efflux Pump Controls Growth, Antibiotic Susceptibility and Virulence in *Pseudomonas aeruginosa* Via 4-Quinolone-Dependent Cell-to-Cell Communication. Microbiology.

[182] Juhas M., Wiehlmann L., Huber B., Jordan D., Lauber J., Salunkhe P., Limpert A.S., von Gotz F., Steinmetz I., Eberl L., Tummler B. (2004). Global Regulation of Quorum Sensing and Virulence by VqsR in *Pseudomonas aeruginosa*. Microbiology.

[183] Ledgham F., Ventre I., Soscia C., Foglino M., Sturgis J.N., Lazdunski A. (2003). Interactions of the Quorum Sensing Regulator QscR: Interaction with itself and the Other Regulators of *Pseudomonas aeruginosa* LasR and RhlR. Mol. Microbiol..

[184] Rampioni G., Bertani I., Zennaro E., Polticelli F., Venturi V., Leoni L. (2006). The Quorum-Sensing Negative Regulator RsaL of *Pseudomonas aeruginosa* Binds to the *lasI* Promoter. J. Bacteriol..

[185] Rampioni G., Polticelli F., Bertani I., Righetti K., Venturi V., Zennaro E., Leoni L. (2007). The *Pseudomonas* Quorum-Sensing Regulator RsaL Belongs to the Tetrahelical Superclass of H-T-H Proteins. J. Bacteriol..

[186] Hentzer M., Wu H., Andersen J.B., Riedel K., Rasmussen T.B., Bagge N., Kumar N., Schembri M.A., Song Z., Kristoffersen P. (2003). Attenuation of *Pseudomonas aeruginosa* Virulence by Quorum Sensing Inhibitors. EMBO J..

[187] Geske G.D., Wezeman R.J., Siegel A.P., Blackwell H.E. (2005). Small Molecule Inhibitors of Bacterial Quorum Sensing and Biofilm Formation. J. Am. Chem. Soc..

[188] Jorgensen F., Bally M., Chapon-Herve V., Michel G., Lazdunski A., Williams P., Stewart G.S. (1999). RpoS-Dependent Stress Tolerance in *Pseudomonas aeruginosa*. Microbiology.

[189] Kojic M., Venturi V. (2001). Regulation of *rpoS* Gene Expression in *Pseudomonas*: Involvement of a TetR Family Regulator. J. Bacteriol..

[190] Whiteley M., Parsek M.R., Greenberg E.P. (2000). Regulation of Quorum Sensing by RpoS in *Pseudomonas aeruginosa*. J. Bacteriol..

[191] Medina G., Juarez K., Soberon-Chavez G. (2003). The *Pseudomonas aeruginosa rhlAB* Operon is Not Expressed during the Logarithmic Phase of Growth Even in the Presence of its Activator RhlR and the Autoinducer *N*-Butyryl-Homoserine Lactone. J. Bacteriol..

[192] Schuster M., Hawkins A.C., Harwood C.S., Greenberg E.P. (2004). The *Pseudomonas aeruginosa* RpoS Regulon and its Relationship to Quorum Sensing. Mol. Microbiol..

[193] Ahmer B.M. (2004). Cell-to-Cell Signalling in *Escherichia coli* and *Salmonella enterica*. Mol. Microbiol..

[194] Van Houdt R., Aertsen A., Moons P., Vanoirbeek K., Michiels C.W. (2006). *N*-Acyl-l-Homoserine Lactone Signal Interception by *Escherichia coli*. FEMS Microbiol. Lett..

[195] De Keersmaecker S.C., Sonck K., Vanderleyden J. (2006). Let LuxS Speak Up in AI-2 Signaling. Trends Microbiol..

[196] Walters M., Sperandio V. (2006). Quorum Sensing in *Escherichia coli* and *Salmonella*. Int. J. Med. Microbiol..

[197] Kanamaru K., Kanamaru K., Tatsuno I., Tobe T., Sasakawa C. (2000). SdiA, an *Escherichia coli* Homologue of Quorum-Sensing Regulators, Controls the Expression of Virulence Factors in Enterohaemorrhagic *Escherichia coli* O157:H7. Mol. Microbiol..

[198] Nealson K.H., Platt T., Hastings J.W. (1970). Cellular Control of the Synthesis and Activity of the Bacterial Luminescent System. J. Bacteriol..

[199] Michael B., Smith J.N., Swift S., Heffron F., Ahmer B.M. (2001). SdiA of *Salmonella enterica* is a LuxR Homolog that Detects Mixed Microbial Communities. J. Bacteriol..

[200] Ravichandiran V., Shanmugam K., Solomon A.P. (2013). Screening of SdiA Inhibitors from Melia Dubia Seeds Extracts Towards the Hold Back of Uropathogenic *E. Coli* Quorum Sensing-Regulated Factors. J. Med. Chem..

[201] Chong T.M., Koh C.L., Sam C.K., Choo Y.M., Yin W.F., Chan K.G. (2012). Characterization of Quorum Sensing and Quorum Quenching Soil Bacteria Isolated from Malaysian Tropical Montane Forest. Sensors.

[202] Bjarnsholt T., Jensen P.O., Rasmussen T.B., Christophersen L., Calum H., Hentzer M., Hougen H.P., Rygaard J., Moser C., Eberl L. (2005). Garlic Blocks Quorum Sensing and Promotes Rapid Clearing of Pulmonary *Pseudomonas aeruginosa* Infections. Microbiology.

[203] Jha B., Kavita K., Westphal J., Hartmann A., Schmitt-Kopplin P. (2013). Quorum Sensing Inhibition by *Asparagopsis. taxiformis*, a Marine Macro Alga: Separation of the Compound that Interrupts Bacterial Communication. Mar. Drugs.

[204] March J.C., Bentley W.E. (2004). Quorum Sensing and Bacterial Cross-Talk in Biotechnology. Curr. Opin. Biotechnol..

[205] Hardie K.R., Cooksley C., Green A.D., Winzer K. (2003). Autoinducer 2 Activity in *Escherichia coli* Culture Supernatants can be Actively Reduced Despite Maintenance of an Active Synthase, LuxS. Microbiology.

[206] Xavier K.B., Bassler B.L. (2005). Regulation of Uptake and Processing of the Quorum-Sensing Autoinducer AI-2 in *Escherichia coli*. J. Bacteriol..

[207] Wang L., Li J., March J.C., Valdes J.J., Bentley W.E. (2005). *luxS*-Dependent Gene Regulation in *Escherichia coli* K-12 Revealed by Genomic Expression Profiling. J. Bacteriol..

[208] Taga M.E., Miller S.T., Bassler B.L. (2003). Lsr-Mediated Transport and Processing of AI-2 in *Salmonella typhimurium*. Mol. Microbiol..

[209] Li J., Attila C., Wang L., Wood T.K., Valdes J.J., Bentley W.E. (2007). Quorum Sensing in *Escherichia coli* is Signaled by AI-2/LsrR: Effects on Small RNA and Biofilm Architecture. J. Bacteriol..

[210] Robinson L.S., Ashman E.M., Hultgren S.J., Chapman M.R. (2006). Secretion of Curli Fibre Subunits is Mediated by the Outer Membrane-Localized CsgG Protein. Mol. Microbiol..

[211] Barnhart M.M., Chapman M.R. (2006). Curli Biogenesis and Function. Ann. Rev. Microbiol..

[212] DeLisa M.P., Wu C.F., Wang L., Valdes J.J., Bentley W.E. (2001). DNA Microarray-Based Identification of Genes Controlled by Autoinducer 2-Stimulated Quorum Sensing in *Escherichia coli*. J. Bacteriol..

[213] Schauder S., Shokat K., Surette M.G., Bassler B.L. (2001). The LuxS Family of Bacterial Autoinducers: Biosynthesis of a Novel Quorum-Sensing Signal Molecule. Mol. Microbiol..

[214] Diaz Z., Xavier K.B., Miller S.T. (2009). The Crystal Structure of the *Escherichia coli* Autoinducer-2 Processing Protein LsrF. PLoS One.

[215] Xavier K.B., Miller S.T., Lu W., Kim J.H., Rabinowitz J., Pelczer I., Semmelhack M.F., Bassler B.L. (2007). Phosphorylation and Processing of the Quorum-Sensing Molecule Autoinducer-2 in Enteric Bacteria. ACS Chem. Biol..

[216] Kendall M.M., Rasko D.A., Sperandio V. (2007). Global Effects of the Cell-to-Cell Signaling Molecules Autoinducer-2, Autoinducer-3, and Epinephrine in a *luxS* Mutant of Enterohemorrhagic *Escherichia coli*. Infect. Immun..

[217] Kaper J.B., Nataro J.P., Mobley H.L. (2004). Pathogenic *Escherichia coli*. Nat. Rev. Microbiol..

[218] Walters M., Sircili M.P., Sperandio V. (2006). AI-3 Synthesis is Not Dependent on *luxS* in *Escherichia coli*. J. Bacteriol..

[219] Sperandio V., Torres A.G., Giron J.A., Kaper J.B. (2001). Quorum Sensing is a Global Regulatory Mechanism in Enterohemorrhagic *Escherichia coli* O157:H7. J. Bacteriol..

[220] Freestone P.P., Lyte M., Neal C.P., Maggs A.F., Haigh R.D., Williams P.H. (2000). The Mammalian Neuroendocrine Hormone Norepinephrine Supplies Iron for Bacterial Growth in the Presence of Transferrin Or Lactoferrin. J. Bacteriol..

[221] Sperandio V., Torres A.G., Kaper J.B. (2002). Quorum Sensing Escherichia Coli Regulators B and C (QseBC): A Novel Two-Component Regulatory System Involved in the Regulation of Flagella and Motility by Quorum Sensing in *E. Coli*. Mol. Microbiol..

[222] Reading N.C., Rasko D.A., Torres A.G., Sperandio V. (2009). The Two-Component System QseEF and the Membrane Protein QseG Link Adrenergic and Stress Sensing to Bacterial Pathogenesis. Proc. Natl. Acad. Sci. USA.

[223] Sperandio V., Li C.C., Kaper J.B. (2002). Quorum-Sensing *Escherichia coli* Regulator A: A Regulator of the LysR Family Involved in the Regulation of the Locus of Enterocyte Effacement Pathogenicity Island in Enterohemorrhagic *E. coli*. Infect. Immun..

[224] Wang D., Ding X., Rather P.N. (2001). Indole can Act as an Extracellular Signal in *Escherichia coli*. J. Bacteriol..

[225] World Health Organization (2014). Antimicrobial Resistance: Global Report on Surveillance 2014.

[226] Ito A., Taniuchi A., May T., Kawata K., Okabe S. (2009). Increased Antibiotic Resistance of *Escherichia coli* in Mature Biofilms. Appl. Environ. Microbiol..

[227] O’Loughlin C.T., Miller L.C., Siryaporn A., Drescher K., Semmelhack M.F., Bassler B.L. (2013). A Quorum-Sensing Inhibitor Blocks *Pseudomonas aeruginosa* Virulence and Biofilm Formation. Proc. Natl. Acad. Sci. USA.

[228] Deep A., Chaudhary U., Gupta V. (2011). Quorum Sensing and Bacterial Pathogenicity: From Molecules to Disease. J. Lab. Phys..

